# Understanding the Genetic Diversity of Picobirnavirus: A Classification Update Based on Phylogenetic and Pairwise Sequence Comparison Approaches

**DOI:** 10.3390/v13081476

**Published:** 2021-07-28

**Authors:** Lester J. Perez, Gavin A. Cloherty, Michael G. Berg

**Affiliations:** Infectious Diseases Research, Abbott Diagnostics, Abbott Park, IL 60064, USA; gavin.cloherty@abbott.com (G.A.C.); michael.berg@abbott.com (M.G.B.)

**Keywords:** picobirnavirus, phylogenetics, pairwise sequence comparison, RdRp, capsid, reassortment

## Abstract

Picobirnaviruses (PBVs) are small, double stranded RNA viruses with an ability to infect a myriad of hosts and possessing a high degree of genetic diversity. PBVs are currently classified into two genogroups based upon classification of a 200 nt sequence of RdRp. We demonstrate here that this phylogenetic marker is saturated, affected by homoplasy, and has high phylogenetic noise, resulting in 34% unsolved topologies. By contrast, full-length RdRp sequences provide reliable topologies that allow ancestralism of members to be correctly inferred. MAFFT alignment and maximum likelihood trees were established as the optimal methods to determine phylogenetic relationships, providing complete resolution of PBV RdRp and capsid taxa, each into three monophyletic groupings. Pairwise distance calculations revealed these lineages represent three species. For RdRp, the application of cutoffs determined by theoretical taxonomic distributions indicates that there are five genotypes in species 1, eight genotypes in species 2, and three genotypes in species 3. Capsids were also divided into three species, but sequences did not segregate into statistically supported subdivisions, indicating that diversity is lower than RdRp. We thus propose the adoption of a new nomenclature to indicate the species of each segment (e.g., PBV-C1R2).

## 1. Introduction

Picobirnaviruses (PBVs) are small, double stranded RNA viruses with two genome segments encoding a capsid and RNA-dependent RNA polymerase (RdRp). PBVs are typically isolated from fecal samples and associated with diarrhea in immunocompromised individuals or as coinfections of gastrointestinal viruses and bacteria [1]. Shedding has also been observed in healthy, asymptomatic individuals or animals and can persist for months [2]. More recently, certain strains have been found in alimentary tracts of mammals and associated with respiratory illnesses [3,4,5,6,7]. PBVs are found in a wide range of geographies and hosts, including vertebrates (e.g., mammals, birds, fish, reptiles, etc.), invertebrates (mollusks, insects, etc.), and in environmental samples like sewage [8]. Identical sequences found in different hosts (e.g., pigs and humans) gives an indication of its facile spread, the absence of species barriers, and the lack of virus–host coevolutionary relationships [4,6]. The possibility has been raised that PBVs actually infect bacteria [9]. Cited as evidence is the inability to culture in mammalian cells, the presence of 5′ Shine–Dalgarno sequences, its relatedness to fungus-infecting partitiviruses, and the usage of an alternate, mitochondrial genetic code by some PBVs [10]. Regardless of what kingdom they truly infect, PBVs exhibit a high degree of genetic diversity [8].

The PBV genus has been divided into two genogroups (G-I and G-II) on the basis of genome segment 2 (RdRp) phylogeny. There has been an attempt to further classify species by virtue of the infected host (e.g., human picobirnavirus, rabbit picobirnavirus, etc.), but given the ability to readily jump from one species to the next, this is clearly inaccurate [11,12]. Likewise, PBV strains derived from various animals are widely distributed throughout the phylogenetic tree, again demonstrating the lack of a continuous spectrum of viruses linked to a particular host [3,4]. The majority of sequences in GenBank have been generated by PCR of a 200/346 nt long fragment in RdRp meant to distinguish G-I and G-II, a sequence spanning nucleotides 665–865 (reference strain 1-CHN-97 of G-I), hereafter denoted as the ‘RdRp-200′ marker [13]. Several groups have noted the inadequacy of this genetic marker [14,15]. The short sequences yield unreliable topologies with low branch supports and unresolved taxa, which typically do not reflect the phylogenetic structures of entire, full length RdRps. Knox et al. [16] compared these amplicons or trimmed sequences to complete genomic sequences, which resulted in incongruent trees that will inevitably lead to incorrect conclusions about evolutionary relationships [16]. Given these shortcomings and the extent of PBV diversity compared to other related dsRNA viruses like Birnaviridae [17], it has been suggested that a formal taxonomic revision needed to be undertaken.

Recent advances in technologies, including phylogenetic methods and associated computational capacity, along with the ability to readily obtain full genomes via metagenomic next generation sequencing (mNGS), can lead to improved classification of groups. Here we continue efforts to establish a consistent phylogenetic methodology to provide a more accurate evolutionary analysis and better understanding of PBV phylogenetic diversity.

## 2. Materials and Methods

### 2.1. Retrieval of Picobirnavirus Sequences

Two sequence datasets (*n* = 2542 sequences) containing all Picobirnavirus (PBV) genus capsid and RdRp coding regions were downloaded from the GenBank database (http://www.ncbi.nlm.nih.gov/) accessed on 1 February 2021. Unique sequences were obtained by filtration according to De la Cruz et al. [18] and deduplication with DAMBE software [19,20], keeping the most ancestral isolate/strain (Tables S1 and S2). Two sequence datasets were prepared containing alignments of the entire coding regions for capsid and RdRp. A third dataset contains a shorter alignment, obtained by trimming the same complete RdRp sequences and retaining the coding region flanked by the RdRp-200 marker, thus including the same representation of genetic diversity and allowing a direct comparison between phylogenetic markers. Additional information was extracted, including year of collection, country, and host. Sequences from ‘PBV-like’ groups were omitted from the dataset for the following three reasons: (i) these sequences are highly divergent from the PBV genus, (ii) they lack a capsid gene, and (iii) most members use an alternative mitochondrial genetic code for translation of their RdRp. The phylogenetic noise introduced by inclusion of this putative genus is expected to increase the uncertainty of the topologies in the PBV genus.

### 2.2. Evaluation of the RdRp-200 Phylogenetic Marker

The evaluation of the quality and reliability of the RdRp-200 marker, commonly used to identify ancestral relationships between nucleotide/amino acids sequences among PBV strains [13] was performed following the methodology developed in Alfonso-Morales et al. [21]. The loss of phylogenetic information due to saturation of substitutions was evaluated by comparing the complete PBV RdRp coding sequence to the 200-RdRp marker. Saturation levels were evaluated by plotting the pairwise number of observed transitions and transversions versus genetic distance. Analysis was performed using the DAMBE software package [19,20] and the results were visualized with GraphPad Prism software 9.0.2 (1992–2021, GraphPad Prism software LLC, San Diego, CA, USA). The information entropy-based index of the substitution saturation approach implemented in DAMBE [19] was used to determine the overall homoplasy signal of the RdRp-200 marker. The consistency of phylogenetic signals derived from sequence datasets of the RdRp-200 marker and the complete coding sequence of RdRp were investigated by likelihood mapping [22]. We generated 100,000 random quartets using TreePuzzle and if more than 30% of the dots fell in the center of the triangle, the data were considered unreliable for phylogenetic inference purposes.

### 2.3. Multiple Sequence Alignment (MSA) and Evaluation of the MSA Accuracy

Nucleotides and amino acid sequences of PBV capsid and RdRp were aligned using the three most common multiple sequence alignment (MSA) methodologies: CLUSTAL W in the BioEdit Sequence Alignment Editor [23], multiple sequence comparison by log-expectation (MUSCLE) software freely available at https://www.ebi.ac.uk/Tools/msa/muscle, accessed on 1 February 2021, and multiple alignment using fast Fourier transform (MAFFT) with the option E-INS-I to decrease the penalty in the gaps. To determine the level of agreement among the different methodologies with an estimated ratio, the accuracy of the alignments was assessed by comparing the measurement of precision determined by the modeler score to the measure of recall determined by the sum of pairs score (SP-score) algorithms implemented in FastSP v. 1.6.0 [24].

### 2.4. Phylogenetic Tree Reconstruction

The impact of the phylogenetic inference on picobirnavirus topology reconstruction was evaluated by two tree-building methodologies: (i): distance-building, e.g., neighbor-joining (NJ) [25] and (ii): character-building, represented by two probabilistic methods including maximum likelihood (ML) [26] for inferring evolutionary nodal connections and Bayesian phylogenetic inference for sampling a priori and posterior distributions of likelihood [27]. Both NJ and BI approaches were computed as described in Perez et al. [28] with some modifications. Detection of recombinant sequences was performed using the RDP5 software package, which includes RDP, Geneconv, MaxChi, Chimaera, and 3Seq methods. Since no evidence of recombination was detected, entire datasets collected after the deduplication process were used for phylogenetic inference analyses (Tables S1 and S2). In addition, amino acids were used as input files and MEGA X [29] was used to compute NJ trees. For BI, the Markov chain Monte Carlo (MCMC) search was run with four chains for 5 million generations, sampling the Markov chain every 100 generations with MrBayes 3.2 software [30]. For ML, the methodology recently described in Pikula et al. [17] was used with some modifications. ML-phylogenetic trees derived from amino acid alignments were computed with the IQ-TREE 2 program [31]. IQ-TREE 2 was also used to select the best-fit model from the analyzed datasets. Confidence levels for branches were determined in IQ-TREE by the Shimodaira test with 10,000 bootstrap replicates and trees were then visualized and edited in FigTree v1.4.3.

### 2.5. Assessing the Reliability of the Phylogenetic Trees by the Comparison of Topologies

To perform multiple hypothesis testing on phylogenetic data, topologies were evaluated by several conventional testing procedures including the Kishino and Hasegawa test (KH) [32], the Shimodaira–Hasegawa test (SH) [33], and the weighted KH and SH (WKH and WSH) tests [34], which compute the log-likelihoods per site for each tree and compare the total log-likelihoods for each proposed topology. In addition, the recently developed approximately unbiased test (AU) [35] and expected likelihood weight test [36] were also conducted using IQ-TREE 2 [31].

### 2.6. Taxonomical Demarcation Analysis Using Pairwise Sequence Comparison (PASC) and Sequence Demarcation Tool (SDT)

Pairwise sequence comparison (PASC) [37] and the recently developed sequence demarcation tool (SDT) [38] were used to assess the levels of taxonomic demarcation within the Picobirnavirus genus for both capsid and RdRp coding regions [39]. A total of 403 unique PBV RdRp and 422 capsid-coding sequences were submitted to the Web tool DIVEIN [40] to obtain PASC mismatch distribution histograms relating divergence/diversity among and within PBV lineages. SDT applies a robust Needleman–Wunsch (NW)-based pairwise alignment approach with a pairwise identity calculation that ignores positions containing indels. It is not restricted to use with predefined sets of sequences, but rather is primarily intended to objectively assign ICTV-endorsed taxonomic classifications of strain, species, and genus based on pairwise identity demarcation thresholds [22]. In parallel, pairwise nucleotide p-distances were calculated using MEGA X [29]. Different matrices of nucleotide/amino acids divergence between groups were generated using 500 bootstrap replicates to estimate variance.

### 2.7. Visualization of Phylogenetic Agreement between RdRp and Capsid and Taxon Sampling Evaluation

Using both ML trees obtained for each PBV genomic segment (coding sequences for RdRp and capsid), a face-to-face phylogenetic tree visualization was computed with an R-script using functions in the R ‘ggtree’ package [41] and tidyverse [42]. Taxon tip aesthetics were interpreted as the PBV-R (PBV-RdRp) and PBV-C (PBV-Capsid) species as defined in the current study and the effects of taxon sampling were evaluated with an R-script running the R ‘tidyverse’ package by using the information from the host, collected in Tables S1 and S2.

## 3. Results

### 3.1. RdRp-200 Marker Is Saturated and Yields Unresolved Topologies

**Figure 1:**
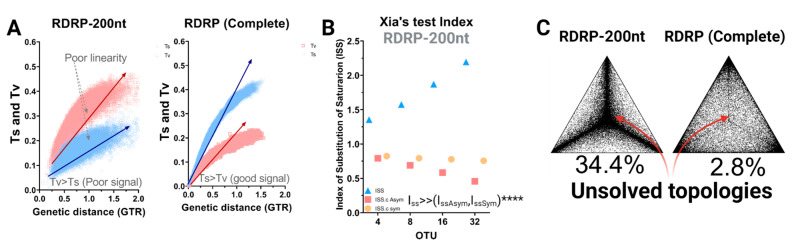
Evaluation of the homoplasy signal and phylogenetic noise. (**A**) Scatter plot representation of transitions and transversions versus the genetic distance calculated using the GTR model implemented in DAMBE [19], comparing RdRp-200 to full-length RdRp. Transition values are denoted with blue circles and transversion values with red squares. (**B**) Xia’s test [20,43] was performed on the 403 sequences for the RdRp-200 marker. DAMBE randomizes sequences in subsets of 4, 8, 16, and 32 OTUs multiple times to perform the test. The index of substitution saturation (ISS), defined as the ratio between the mean entropy of aligned sequences (H) versus the entropy of sequences assuming full saturation (HFSS), is represented by blue triangles (see expression (2), (3), and (5) in Xia et al.) [20]. The index of the critical substitution saturation (ISS.c), defined as the value at which the sequences will begin to fail to recover the true topologies for both symmetric and asymmetric trees, are represented by orange circles and red rectangles, respectively, (**** significant differences *p* < 0.0001). (**C**) Evaluation of phylogenetic noise by maximum-likelihood mapping for both RdRp-200 and RdRp-complete phylogenetic markers. Dots located at triangle vertices represent posterior probabilities of unrooted topologies in each quartet; the percentage of unsolved topologies noted for each corresponds to an estimation of phylogenetic noise.

A starting point in the evaluation of the genetic relationships established by different viral strains is to determine the reliability of the tree topology and the accuracy of the different markers used to yield the inferred tree structure. We started by evaluating three major aspects including saturation, homoplasy, and phylogenetic noise in the most common phylogenetic marker in RdRp used to resolve PBV phylogeny. First, an assessment of saturation of substitution was performed by plotting the absolute number of transitions and transversions versus genetic distance, comparing the commonly used 200-RdRp marker (Figure 1A). For the RdRp-200 marker, the number of transversions (e.g., A→T) relative to transitions (e.g., A→G) displayed an inverted pattern, indicating non-conserved changes outweighed conserved changes and are thus likely to alter the amino acid sequence. The asymptotic trend and dispersion of the data (poor linearity) further suggested a drive towards saturation in RdRp-200, which is indicative of poor phylogenetic resolution (Figure 1A). By contrast, the number of transitions was higher than transversions for the complete coding RdRp sequences. The expected ratio of 2:1–3:1 for a coding region was observed, along with a tighter, linear distribution. Moreover, the number of observed transversions relative to transitions gradually increased with growing divergence, as would be expected (Figure 1A).

Homoplasy leads to incomprehensible evolutionary history by overestimating genetic similarity [44]. A large proportion of homoplasies in a sequence dataset is therefore prone to biasing the inference of phylogenetic relationships. Saturation of substitutions graphically describes the distribution of homoplasy but does not include the consistency index [45]. The information entropy-based index of the substitution saturation approach developed by Xia et al. [20] implements two major approaches to address homoplasy: (i) tree-independent measures based on relative apparent synapomorphy and (ii) the parsimony method proposed specifically to alleviate the problem of sequence convergence due to similarity in nucleotide frequencies [43]. Xia’s test was performed here to estimate homoplasy and provide statistically supported evidence of saturation throughout the RdRp-200 marker (Figure 1B). The index of substitution saturation (Iss) represents the ratio of the mean entropy of aligned sequences of length 200 nt (H) to the entropy of sequences assuming full saturation (HFSS; blue triangles). The index of critical substitution saturation (Iss.c) represents the value at which the sequences will fail to produce accurate topologies for symmetric and asymmetric trees (orange and red circles). Here we observe that the theoretical index of substitution saturation values exceed critical values (Iss >> IsscAsym, IsscSym): the opposite is necessary to obtain reliable topologies. Once again, we demonstrate that this marker is unsuitable for phylogenetic relationship inference and will inevitably lead to incorrect hypotheses of grouping resolutions.

Next, we evaluated phylogenetic noise using maximum likelihood mapping. The phylogenetic quartet is the fundamental unit of the tree graph for which levels exceeding 30% equate to noise. For the RdRp-200 marker, we observed a large percentage of dots (taxa) grouping in the vertices of the triangles such that 34.4% of topologies remained unsolved (central dots) (Figure 1C). With >1/3 of taxa failing to form an inherent quartet structure, this indicates the use of this region as a phylogenetic marker will lead to unreliable topologies. However, using the full-length RdRp, only 2.8% of taxa fall within the central region, which contains unresolved topologies. Therefore, we abandoned the RdRp-200 marker and focused on selecting the best method to analyze full-length RdRp.

### 3.2. Complete Resolution of Taxa Requires Proper Selection of Alignment and Phylogenetic Reconstruction Methods

**Figure 2:**
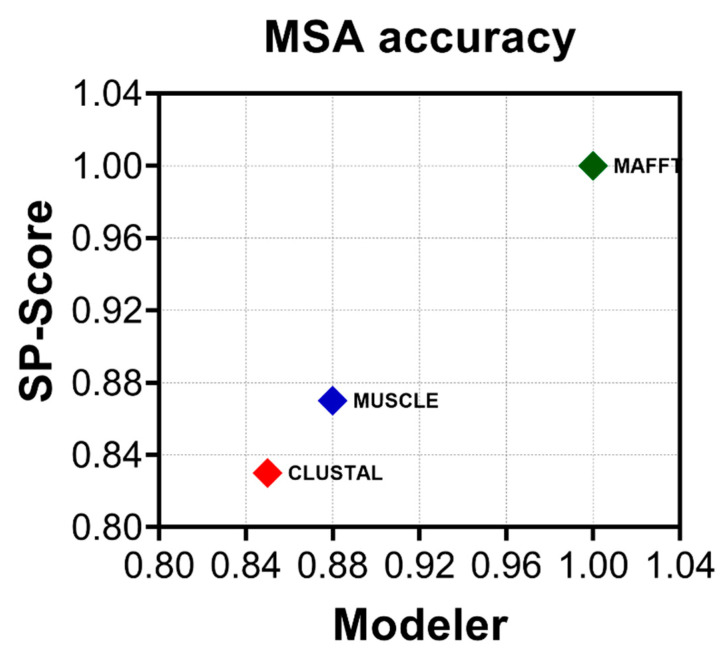
MAFFT provides the most accurate alignment. The average modeler score (precision) versus sum of pairs score (SP-score) (recall) for all three methods used to align the dataset: CLUSTALW (red), MUSCLE (blue), and MAFFT E-INS-i (green). Estimations were performed using a linear time calculation of alignment accuracy (FASTSP) [24].

Multiple sequence alignment (MSA) is a fundamental first step in phylogenetic estimations and as such, alignment errors can lead to significant bias during topology reconstruction [46,47]. The Picobirnavirus genus is highly genetically divergent [16] and while multiple sequence alignment (MSA) using either codons or amino acids has been recommended by different research groups [48,49], the impact of different MSA strategies on topology resolution has yet to be explored. Determining the most accurate method of alignment is dataset-dependent and therefore critical to avoid misinterpretation of downstream analyses [50]. A common methodology to explore accuracy and the agreement between different methods is the estimation of the ratio between the modeler score (as a measure of precision) and the sum of pairs score (SP-score) (as a measure of recall). Sequences aligned with CLUSTAL-W were the least accurate whereas those aligned with MAFFT E-INS-I were the most accurate (Figure 2). We were thus able to evaluate the impact of each MSA on the tree topologies. We obtained 403 complete picobirnavirus RdRp sequences from GenBank and compared the maximum likelihood tree (ML) topologies resulting from an MSA performed with CLUSTAL W, MUSCLE, and MAFFT programs [14,16] (Table 1). The maximal log likelihood (logL) value was calculated for each tree relative to the MSA under investigation using SSP and the modeler. The statistical tests run includes 100,000 resamplings with the RELL method [32], Kishino–Hasegawa test [32], Shimodaira–Hasegawa test [33], expected likelihood weight [36], and the approximately unbiased (AU) test [34]. MAFFT yielded the lowest logL value at −174,747.1905 and was selected as the aligner of choice. Therefore, MAFFT produced the most accurate alignment and it also yielded the best supported topology.

**Figure 3:**
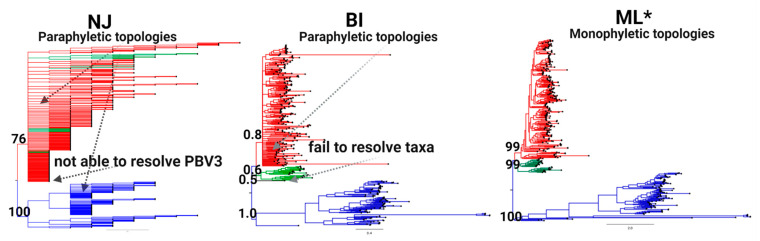
Phylogenetic tree topology comparison for classification of PBV. Tree topologies of the RdRp complete coding sequences obtained by neighbor joining (NJ; left), Bayesian inference (BI; middle), and maximum likelihood (ML; right) analyses are shown. Three main PBV lineages resolved are denoted by color (PBV1: red, PBV2: blue, and PBV3: green) with support for external nodes indicated by bootstrap/posterior probability values. Characteristics (monophyletic or paraphyletic) of each tree are indicated and the best topology supported by all statistical methods deployed (see the Materials and Methods) is denoted with an asterisk. Refer to Table 2 for the results of the tests.

Phylogenetic tree methods were then evaluated with the MAFFT alignment deploying the same battery of statistical tests (Table 2). ML yielded the lowest logL value at −168,943.427 and the optimal topology by all the methodologies deployed, including the well-established KH and SH methods (Table 2). Visual inspection of neighbor-joining (NJ) and Bayesian inference (BI) results detected paraphyletic topologies throughout trees with both BI and NJ failing to resolve a third monophyletic lineage. By contrast, ML trees produced monophyletic topologies across the entire tree and resolved a third distinct lineage supported by a bootstrap value ≥ 99% (Figure 3).

### 3.3. RdRp Lineages Delimit Three Distinctive Species for the Picobirnavirus Genus

**Figure 4:**
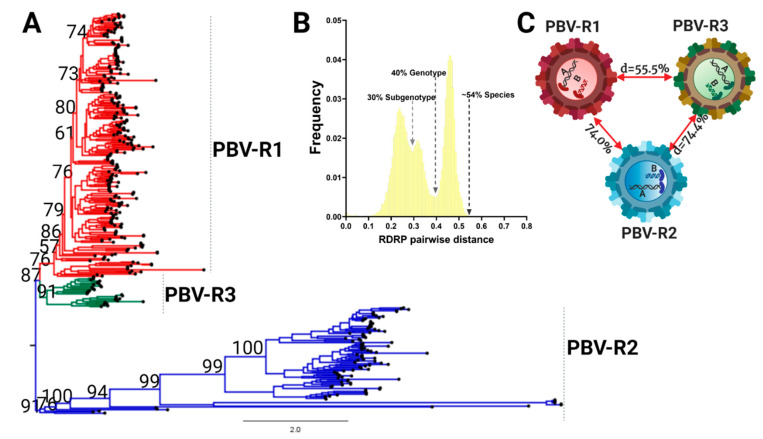
The Picobirnavirus genus differentiates into three species based on the RdRp coding region. (**A**) Phylogenetic tree based on the complete coding sequence of RdRp for all 403 non-redundant genomes available at GenBank and the maximum likelihood (ML) method using the LG + F + R10 model. The main PBV lineages were given the following designations and colors: PBVR1, red; PBVR2, blue; PBVR3, green). All the nodes with statistical support determined by bootstrap values higher than 60% are shown. (**B**) PASC results for the frequency distribution of pairwise distances for all 403 RdRp sequences. Cut-off values for each minor and major division is indicated, with a subgenotype at 30%, genotype at 40%, and different species at 54%. (**C**) Genetic distances for all three main lineages.

Current PBV nomenclature refers to only two genogroups based upon RdRp. Refseq strains widely recognized as GG-I and GG-II reside within PBV-R1 and PBV-R2 lineages defined here (Figure 4A). PBV-R3 sequences were previously contained within genogroup I (PBV-R1), however, the inclusion of new, full length sequences together with the most appropriate methodologies allowed resolution of this lineage. The pairwise sequence comparison (PASC) tool was used to determine Picobirnavirus taxonomic classification based on genomic demarcation. PASC is a widely accepted method in virology for classification purposes that plots the pairwise differences between sequences, also known as the mismatch distribution [22]. An empirically determined distribution of pairwise distances versus frequency for all 403 RdRp complete open reading frames resulted in a distribution characterized by three peaks (representing taxonomically associated groups) and two valleys (representing taxonomic classification cut-offs) (Figure 4B). The profile revealed a minor division of clustering at a threshold of 30% divergence, another medium level demarcation at 40%, with the final peak and whole distribution representing a 54% genetic difference. The pattern was consistent whether we used nucleotides with the DIVEIN tool or amino acids with the sequence demarcation tool (SDT) (Figure S1). To determine the taxonomic level at which the three main lineages resolve in the ML topology, genetic distances between groups were estimated using a p-distance method implemented in MEGA. Genetic inference calculations revealed divergence between PBV-R1 and PBV-R3 was 55.5%, while PBV-R2 diverged further from both at 74.0% and 74.4%, respectively (Figure 4C). With the upper limit of genetic distance for the final division at 54%, less than any of the distances between groups (e.g., >55%), this result revealed that the three lineages were in fact different species. Note there was only one contiguous PASC distribution: two separate distributions would have indicated these were different genera. Thus, taking into consideration the monophyletic branching within each PBV species, it can be inferred that the peak spanning 40–54% genetic differences corresponds to a genotype demarcation, while the 3–40% interval corresponds to a subgenotype grouping.

**Figure 5:**
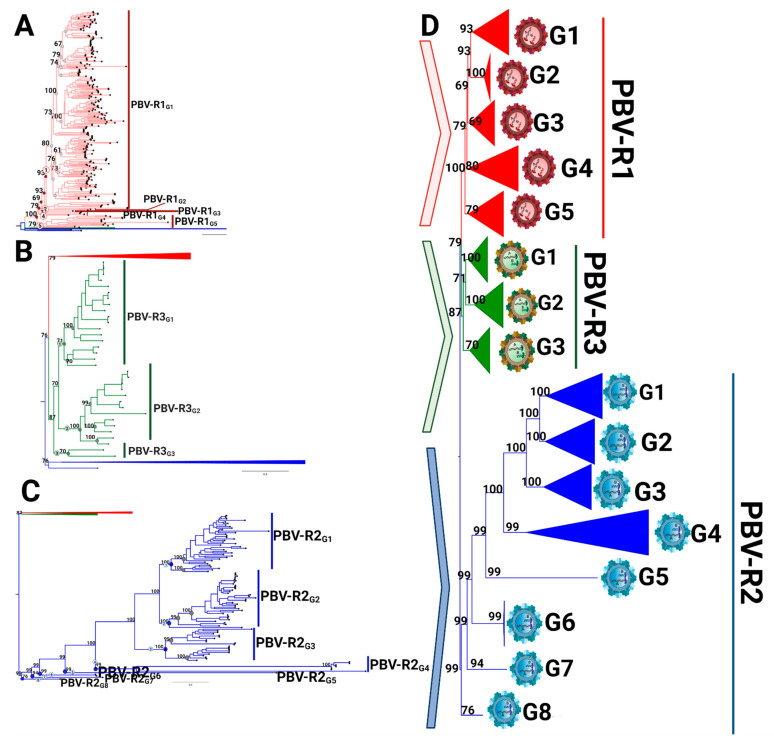
Intraspecies diversity of picobirnavirus based on coding RdRp region. The starting phylogenetic tree is based on the complete coding sequence of RdRp using all 403 non-redundant genomes available at GenBank by the maximum likelihood (ML) method and a WAG + F + R10 model, with each analysis according to the species. (**A**) PBV-R1 sequences are denoted in red, with PBV-R2 and PBV-R3 collapsed to improve the visualization. Nodes of lineages measured are denoted with circles in the tree (left panel): those with genetic distances lower than the cut-off value (40%) are in grey and those above are in red. (**B**) PBV-R2 species sequences are described as in 5A, with PBV-R1 and PBV-R3 collapsed to improve visualization. (**C**) PBV-R3 species sequences are described as in 5A, with PBV-R1 and PBV-R2 collapsed to improve visualization. (**D**) Summary representation of PBV classifications based on RdRp sequences. PASC cut-off values from the previous analysis (see Figure 4B) were used to determine genotype demarcation (values higher than 30% and lower than 40% of genetic distance were considered). The genetic distances estimated used to determine the genotype classification for each species are available in Tables S3–S5.

Using the threshold values determined by PASC analysis (Figure 4B), subdivisions of each species were explored further. Within PBV-R1 we identified 12 statistically supported internal nodes (bootstrap values ≥ 60%; Figure 5A, Table S3), in which genetic distances between them only exceeded 40% for five groupings. Therefore, we defined these five groups as genotypes G1-G5: they are denoted with red dots on the tree, correspond to the red peak in the PASC distribution, and have red cells in the adjacent table (Figure 5A). The remaining seven clusters with pairwise distances below this cutoff (depicted in grey) could constitute subgenotypes, however, we did not pursue further classification at this taxonomic level. We highlight the fact that the branching pattern for PBV-R1G1 exhibits a higher diversity than the remaining genotypes within the PBVR1 species. PBV-R2 and PBV-R3 species were similarly assessed to determine divergence patterns. For PBV-R2, 8 genotypes (denoted in blue) out of 11 groups were identified, while for PBV-R3 only 3 genotypes (denoted in green) out of 6 groups were recognized (Figure 5B,C, Tables S4 and S5). The proposal for the classification of PBV RdRp is summarized in Figure 5D.

### 3.4. Capsid Lineages Also Diverged into Three Distinct Species

**Figure 6:**
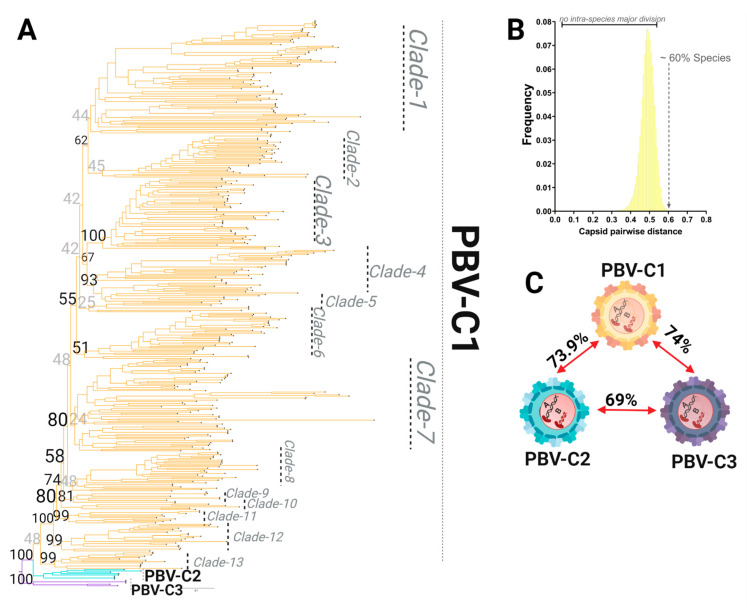
Species demarcation and intraspecies diversity of the picobirnavirus based on the capsid coding region. (**A**) Phylogenetic tree based on the complete coding sequence of capsid using all 422 non-redundant genomes available in GenBank using the maximum likelihood (ML) method with the LG + F + R10 model. (**B**) PASC results for the frequency distribution of pairwise distances for all 422 capsid sequences. The lack of a major division(s) indicates no relevant subclassification of sequences is present. The cut-off value for species demarcation at >60% genetic distance is indicated. (**C**) Genetic distances for the three main lineages are shown. All have >60% genetic distance and thus are considered species, with further internal divisions designated as clades when statistical support for the node and the number of taxa (>3) allowed this classification (see 6A for PBV-C1).

PASC analysis was then applied to 422 capsid sequences following ML tree reconstruction (Figure 6A). As with RdRp, we observed three major capsid lineages obtained from the ML topology using MAFFT alignment (for the best topology yielded see Table 3 and Table 4). However, unlike RdRp, the majority of capsid sequences populated one lineage of the phylogenetic tree, denoted here as PBV-C1. In addition, the PASC analysis distribution was characterized by only one peak, with pairwise genetic distances ranging from 40–60% (Figure 6B; Supplemental Figure S2). This pattern illustrates the lack of internal taxonomical division. Sequences were grouped based on the lineages derived from the phylogenetic tree in order to calculate genetic distances. With genetic distances higher than 60% obtained for all the three main lineages, it was again revealed with the capsid segment that the PBV genus has diversified into three different species. We denote these here as PBVC1-PBVC3 (Figure 6). PASC distribution and the current number of capsid sequences available did not support further classification at the level of the genotype or subgenotype. Thus, the phylogenetic divisions observed within the PBV species C1 (PBVC1) were defined as clades, with a total of 13 clades identified in the current dataset (Figure 6A).

## 4. Discussion

Our rigorous analysis demonstrates the need to standardize phylogenetic methodologies. Starting with the choice of genetic markers, we show clearly that the information contained within the 200 nt region of RdRp was insufficient to reliably distinguish groups. Transversions outnumbered transitions, mutations saturated the length of the sequences, and more than 1/3 of taxa remained unresolved. Trees derived from full-length sequences remedied all these shortcomings and in turn provided new biological insights into the evolution of PBV. The proper combination of the alignment algorithm (MAFFT) and phylogenetic tree reconstruction (maximum likelihood) also avoided bias and achieved greater resolution of taxa. Adopting a uniform set of analysis criteria is imperative so that different research groups are evaluating picobirnavirus sequences in the same manner.

PBV has not been isolated in culture, nor are there serotypes, etc., to distinguish strains. The International Committee on the Taxonomy of Viruses (ICTV) has endorsed, among other phylogenetic and biological criteria, the use of genome-wide nucleotide or amino acid sequence identity thresholds for the classification of novel virus isolates.. Complete RdRp sequences segregated into three lineages, which based upon pairwise genetic distances, we determined are unique species of PBV. The idea that PBVs genogroups I and II diverged into different genera has been suggested in recent studies [3,4,16], however, our results rejected this hypothesis. The frequency of pairwise distributions obtained for both segments using all the complete sequences available at the GenBank revealed a homogeneous distribution, lacking additional peak(s) at greater genetic distances that would indicate the differentiation of PBVs at this major taxonomic level [22,39,51]. PBV-like strains utilizing the mitochondrial genetic code may be from another genus, however, we excluded these from our analysis [10,48]. Future studies are necessary to determine where these fit on the evolutionary tree. PASC analysis further classified taxa within these species into multiple genotypes and subgenotypes. While prior classification into two genogroups was warranted based on the genetic distances calculated across the 200 nt RdRp segment, we demonstrated unequivocally that this marker is under homoplasy and should be avoided. The majority of RdRp sequences are found in PBV-R1, yet the greatest genetic diversity is found in PBV-R2 with eight genotypes. We note that PBV-R3 now resolves from PBV-R1 when using full-length sequences, a pattern that has been demonstrated but not described by others [52]. In spite of this speciation pattern, there does not appear to be evidence of virus–host coevolution or a host restriction at any classification level.

**Figure 7:**
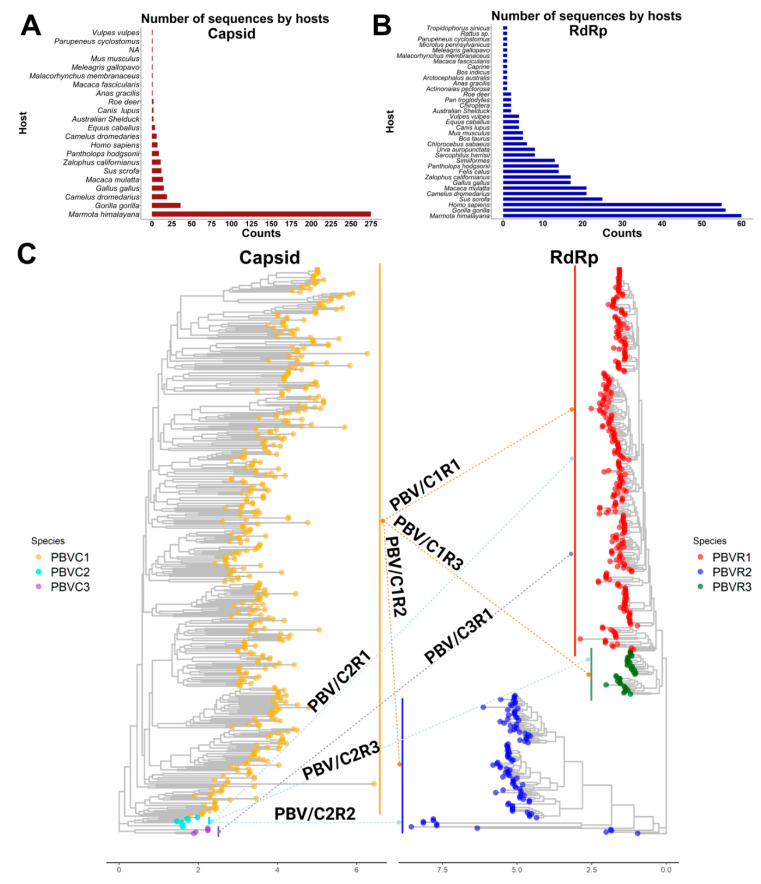
Phylogenetic structure of PBV reassortments observed in nature. (**A**) Plot totaling the number of PBV capsid sequences available in GenBank by host. (**B**) Plot totaling the number of PBV RdRp sequences available in GenBank by host. (**C**) Capsid and RdRp segments are each classified into 3 species. Phylogenetic trees with arrows show segment connections resulting from genetic reassortment for sequences in which both segments are reported in GenBank.

Full-length capsids were also classified into three species, however PASC analysis indicates there were not statistically supported subdivisions therein, only clades in PBV-C1. The lack of observed genetic diversity in capsid may be a reflection of incomplete or biased reporting. Indeed, the vast majority of capsid sequences in GenBank are from Marmota himalayana, whereas for RdRp there is a greater balance of vertebrate entries (Figure 7A,B). An alternative hypothesis is that host selective pressure has placed limits on diversification, ensuring a broadness that accommodates encapsidation of any RdRp while permitting viral entry into a myriad of diverse hosts [53]. For these reasons, we speculated that the capsid segment was acquired later than RdRp following independent reassortment events.

We urge the adoption of the phylogenetic classification scheme and nomenclature set forth here. As with Influenza A virus (e.g., H1-18N1-11) and more recently suggested for infectious bursal disease virus (IBDV), a Birnavirus member (e.g., A0-8B1-5), we suggest picobirnaviruses ought to be named according to their capsid and RdRp species (e.g., C1-3R1-3). Figure 7C summarizes which combinations are observed in nature: most, but not all reassortments are possible. Establishing a greater understanding of the genetic diversification of the PBV genus will allow us to comprehend evolutionary dynamics, better predict ‘host-jumping’ events, and determine which viruses may cause disease in a specific host. Members of the Pestivirus genus, which are genetically and antigenically related and share a broad host-range, illustrate how species demarcations can lead to insights on viral pathogenesis and the application of appropriate control measures, including the development of efficient vaccines and reliable diagnostics [54]. For example, bovine viral diarrhea virus (BVDV) can infect pigs and cause clinical signs (e.g., respiratory, weight loss, anemia, delayed development, congenital tremors (CT), and petechiae on the skin among others) indistinguishable from the classical swine fever virus (CSFV) [55]. However, until the differentiation of CSFV from BVDV as distinct species, their vast differences in virulence and destructive implications for the pig industry were not appreciated. Whereas BVDV infection poses a lesser threat without restriction for herd movement, CSFV can spread rapidly in pigs and cause high mortality, requiring stringent sanitary protocols, restriction of movement/export, vaccinations, and culling of herds [56,57]. While there is no evidence for a host–species demarcation in PBV, our results provide a solid future basis for determining whether certain strains of this genus are linked to specific clinical condition in a given host.

As questions persist regarding whether PBVs are bacterial or vertebrate viruses, a separate debate continues as to whether they are disease-causing, strictly opportunistic infections, or simply a bystander [9,58]. It does yet not appear that arrangements of segments from these PBV species is predictive of different clinical outcomes. However, having established an association with watery diarrhea and more recently with respiratory disease, combined with its ability to rapidly reassort and jump species, picobirnaviruses ought to be cause for greater investigation and concern [5,6,7,59].

## 5. Conclusions

We established that the previously accepted classification of PBV with a 200 nt region of RdRp needs to be replaced by an analysis of full-length sequences using MAFFT alignments and maximum likelihood phylogenetic tree reconstruction. RdRp and capsid segments were each found to have diverged into three species, independent of host and with an ability to freely reassort. Agreed upon methodologies, taxonomic assignments, and nomenclature will further the field’s understanding of PBV evolutionary dynamics and whether emerging strains have the potential to cause disease.

## Figures and Tables

**Table 1:** Topology comparison for different alignment methods for the RdRp gene of PBVs using all 403 complete gene sequences available at the GenBank database. (Best topology detected is highlighted in bold).

Alignment	Tree	logL	deltaL	bp-RELL	p-KH	p-SH	p-WKH	p-WSH	c-ELW	p-AU
Mafft	Clustal_ML	−174,955.2386	208.05	0.007-	0.008-	0.023-	0.008-	0.018-	0.00664-	0.00499-
	Muscle_ML	−175,110.1153	362.92	0-	0-	0-	0-	0-	6.55 × 10^−24^-	0.000423-
	**Mafft_ML**	**−174,747.1905**	**0**	**0.993+**	**0.992+**	**1+**	**0.992+**	**0.999+**	**0.993+**	**0.995+**
Muscle	Clustal_ML	−190,144.7609	591.07	0-	0-	0-	0-	0-	5.17 × 10^−97^-	0.00017-
	Muscle_ML	−189,553.6898	0	1+	1+	1+	1+	1+	1+	0.999+
	Mafft_ML	−189,976.8135	423.12	0-	0-	0-	0-	0-	9.07 × 10^−5^-	0.00115-
Clustal	Clustal_ML	−179,040.1775	0	1+	0.999+	1+	0.999+	1+	1+	1+
	Muscle_ML	−179,564.8764	524.7	0-	0-	0-	0-	0-	5.11 × 10^−94^-	0.00129-
	Mafft_ML	−179,438.5019	398.32	0-	0.001-	0.001-	0.001-	0.002-	1.44 × 10^−12^-	2.68 × 10^−5^-

deltaL: logL difference from the maximal logL in the set. bp-RELL: bootstrap proportion using the RELL method [1]. p-KH: *p*-value of the one sided Kishino–Hasegawa test [1]. p-SH: *p*-value of the Shimodaira–Hasegawa test [2]. p-WKH: *p*-value of the weighted KH test. p-WSH: *p*-value of the weighted SH test. c-ELW: expected likelihood weight [3]. p-AU: *p*-value of the approximately unbiased (AU) test [4]. Plus signs denote the 95% confidence sets. Minus signs denote significant exclusion. All tests performed 100,000 resamplings using the RELL method.

**Table 2:** Topology comparison for phylogenetic methods for the RdRp gene of PBVs using all 403 complete gene sequences available at the GenBank database. (Best topology detected is highlighted in bold).

Alignment	Tree	logL	deltaL	bp-RELL	p-KH	p-SH	p-WKH	p-WSH	c-ELW	p-AU
Mafft	NJ	−170,984.6633	2041.2	0-	0-	0-	0-	0-	0-	1.92 × 10^−9^-
**Mafft**	**ML**	**−168,943.427**	**0**	**1+**	**1+**	**1+**	**1+**	**1+**	**1+**	**1+**
Mafft	BI	−172,399.3318	3455.9	0-	0-	0-	0-	0-	0-	3.66 × 10^−42^-

NJ: neighbor joining method; ML: maximum likelihood; BI: Bayesian inference; deltaL: logL difference from the maximal logL in the set. bp-RELL: bootstrap proportion using the RELL method [1]. p-KH: *p*-value of the one sided Kishino–Hasegawa test [1]. p-SH: *p*-value of the Shimodaira–Hasegawa test [2]. p-WKH: *p*-value of the weighted KH test. p-WSH: *p*-value of the weighted SH test. c-ELW: expected likelihood weight [3]. p-AU: *p*-value of the approximately unbiased (AU) test [4]. Plus signs denote the 95% confidence sets. Minus signs denote significant exclusion. All tests performed 100,000 resamplings using the RELL method.

**Table 3:** Topology comparison for phylogenetic methods for the capsid gene of PBVs using all 422 complete gene sequences available at the GenBank database. (Best topology detected is highlighted in bold).

Alignment	Tree	logL	deltaL	bp-RELL	p-KH	p-SH	p-WKH	p-WSH	c-ELW	p-AU
Mafft	Clustal_ML	−398,083.4813	500.35	0-	0-	0-	0-	0-	9.57 × 10^−10^-	3.6 × 10^−5^-
	**Mafft_ML**	**−397,583.133**	**0**	**0.996+**	**0.994+**	**1+**	**0.994+**	**0.999+**	**0.996+**	**0.994+**
	Muscle_ML	−397,870.1183	286.99	0.0039-	0.0056-	0.0107-	0.0056-	0.0109-	0.00394-	0.00696-
Muscle	Clustal_ML	−433,375.5232	628.32	0-	0-	0-	0-	0-	1.49 × 10^−89^-	0.00124-
	Mafft_ML	−433,115.9208	368.71	0.0002-	0.0002-	0.0005-	0.0002-	0.0005-	0.000204-	0.000896-
	Muscle_ML	−432,747.2061	0	1+	1+	1+	1+	1+	1+	0.999+
Clustal	Clustal_ML	−411,397.5066	0	1+	1+	1+	1+	1+	1+	1+
	Mafft_ML	−412,096.1356	698.63	0-	0-	0-	0-	0-	1.9 × 10^−101^-	3 × 10^−8^-
	Muscle_ML	−412,145.092	747.59	0-	0-	0-	0-	0-	3.7 × 10^−144^-	0.00208-

deltaL: logL difference from the maximal logL in the set. bp-RELL: bootstrap proportion using the RELL method [32]. p-KH: *p*-value of the one sided Kishino–Hasegawa test [32]. p-SH: *p*-value of the Shimodaira–Hasegawa test [33]. p-WKH: *p*-value of the weighted KH test. p-WSH: *p*-value of the weighted SH test. c-ELW: expected likelihood weight [36]. *p*-AU: *p*-value of the approximately unbiased (AU) test [35]. Plus signs denote the 95% confidence sets. Minus signs denote significant exclusion. All tests performed 100,000 resamplings using the RELL method.

**Table 4:** Topology comparison for the phylogenetic methods for the capsid gene of PBVs using all 422 complete gene sequences available at the GenBank database. (Best topology detected is highlighted in bold).

Alignment	Tree	logL	deltaL	bp-RELL	p-KH	p-SH	p-WKH	p-WSH	c-ELW	p-AU
Mafft	NJ	−392,286.036	768.5	0-	0-	0-	0-	0-	1.06 × 10^−146^-	5.05 × 10^−42^-
**Mafft**	**ML**	**−389,942.269**	**0**	**1+**	**1+**	**1+**	**1+**	**1+**	**1+**	**1+**
Mafft	BI	−392,286.036	2343.8	0-	0-	0-	0-	0-	0-	1.65 × 10^−42^-

deltaL: logL difference from the maximal logL in the set. bp-RELL: bootstrap proportion using the RELL method [32]. p-KH: *p*-value of the one sided Kishino–Hasegawa test [32]. p-SH: *p*-value of the Shimodaira–Hasegawa test [33]. p-WKH: *p*-value of the weighted KH test. p-WSH: *p*-value of the weighted SH test. c-ELW: expected likelihood weight [36]. p-AU: *p*-value of the approximately unbiased (AU) test [35]. Plus signs denote the 95% confidence sets. Minus signs denote significant exclusion. All tests performed 100,000 resamplings using the RELL method.

## Data Availability

All the raw data including multiple sequence alignments and phylogenetic trees obtained during the execution of the current study are available in the public repository below. The R-scripts used for data visualization and analyzing the effects of taxon sampling are also available in the same repository: https://github.com/LesterJP/PBV_Research.

## Supplementary Materials

The following are available online at https://www.mdpi.com/article/10.3390/v13081476/s1, Figure S1. Frequency distribution of pairwise distance and clustering pattern for all lineages of PBV for RdRp, Figure S2. Frequency distribution of pairwise distance and clustering pattern for all lineages of PBV for capsid, Supplemental Table S1. Listing of GenBank RDRP references, Supplemental Table S2. Listing of GenBank Capsid references, Table S3. Genetic distances based on complete RDRP coding sequences of all the lineages assessed within the PBV species 1, Table S4. Genetic distances based on complete RDRP coding sequences of all the lineages assessed within the PBV species 2, Table S5. Genetic distances based on complete RDRP coding sequences of all the lineages assessed within the PBV species 3.
