# Supplementary material for: Understanding the Genetic Diversity of Picobirnavirus: A Classification Update Based on Phylogenetic and Pairwise Sequence Comparison Approaches
Source: Viruses. 2021 Jul 28;13(8):1476. doi: 10.3390/v13081476 (PMC8402817; doi:10.3390/v13081476)
Supplement: Supplementary file 1 [file viruses-13-01476-s001.zip › viruses-1294990-supplementary.pdf]

# Online Supplemental Information

Understanding the genetic diversity of Picobirnavirus: a classification update based on phylogenetic and pairwise sequence comparison approaches.

Lester J. Perez, Michael G. Berg, Gavin A. Cloherty
Infectious Diseases Research, Abbott Diagnostics, Abbott Park IL

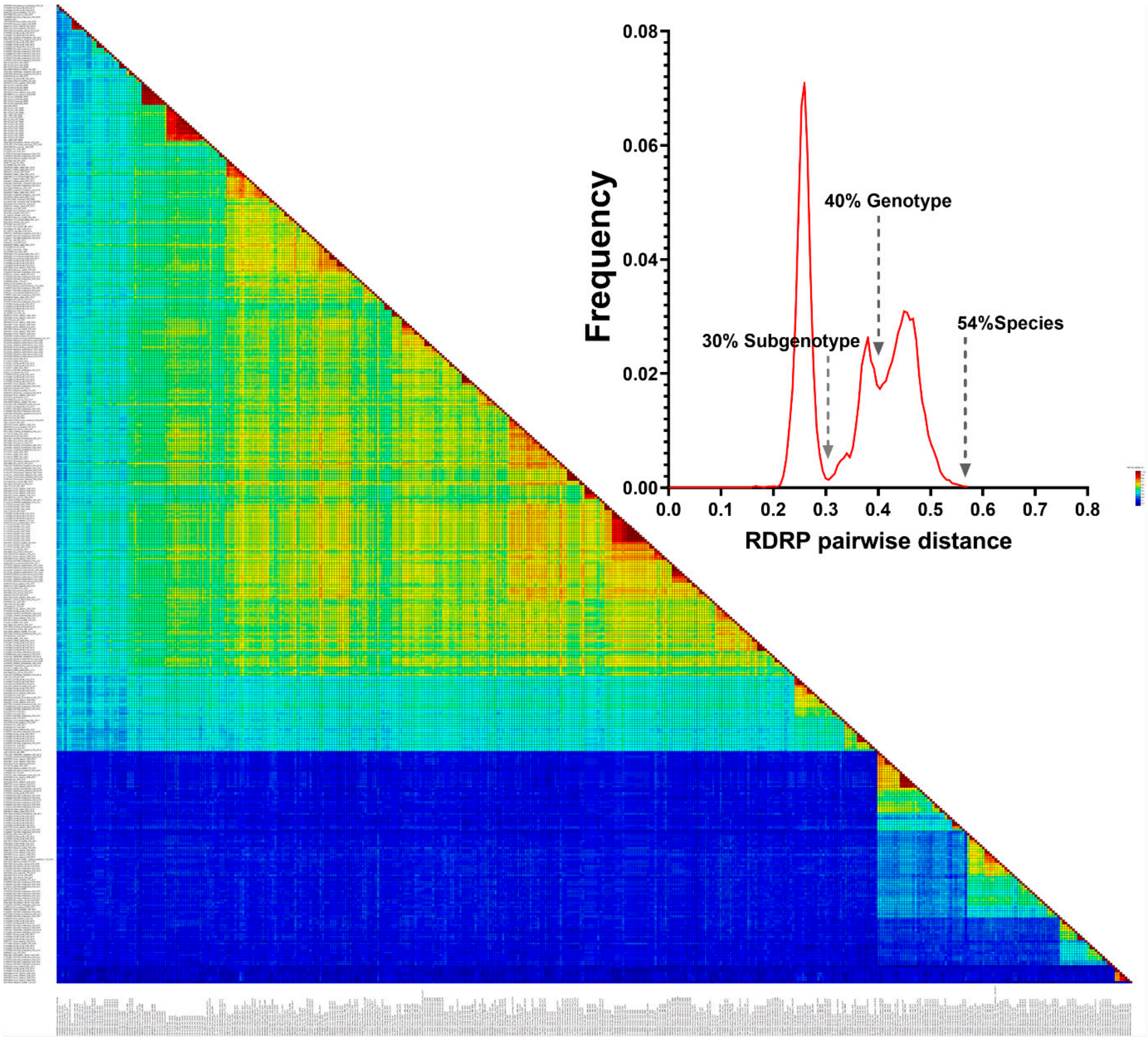


**Figure S1. Frequency distribution of pairwise distance and clustering pattern for all lineages of PBV for RdRp.** Results obtained from SDT represented by a color-coded pairwise identity matrix generated from the 403 RdRp complete coding sequences of PBV included in the current study. Each colored cell represents a percentage of identity score between two sequences (one indicated horizontally to the left and the other vertically at the bottom). A colored key indicates the correspondence between pairwise identities and the colors displayed in the matrix. Pairwise identity frequency distribution plot is also shown. The horizontal axis indicates percentage pairwise distance. The cut-off values for sub-genotype, genotype and species were also denoted.

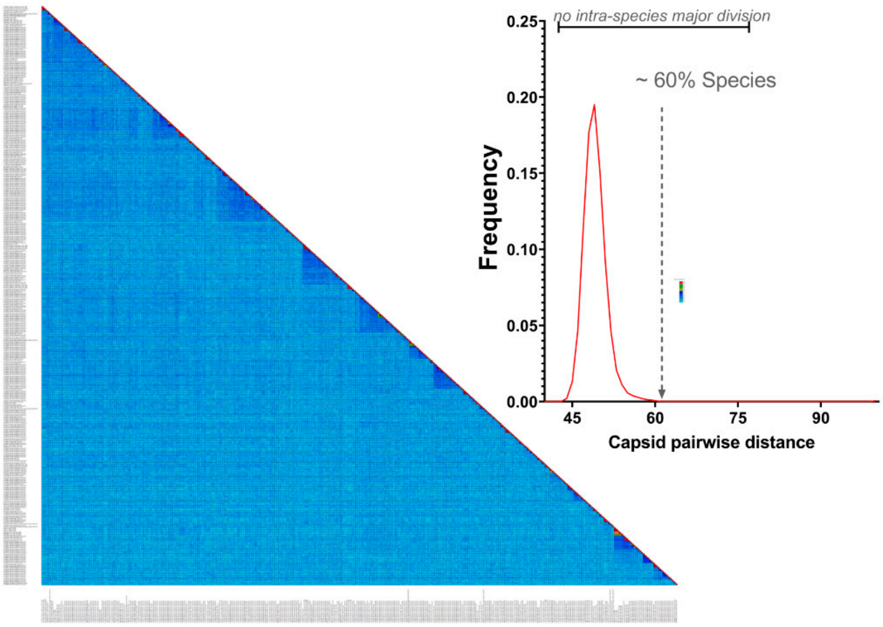


**Figure S2. Frequency distribution of pairwise distance and clustering pattern for all lineages of PBV for capsid.** Results obtained from SDT represented by a color-coded pairwise identity matrix generated from the 422 complete capsid coding sequences of PBV included in the current study. Each colored cell represents a percentage of identity score between two sequences (one indicated horizontally to the left and the other vertically at the bottom). A colored key indicates the correspondence between pairwise identities and the colors displayed in the matrix. Pairwise identity frequency distribution plot is also shown. The horizontal axis indicates percentage pairwise distance. The lack of intra-species division and the cut-off value for species demarcation are denoted.

**Supplemental Table S1.** Listing of GenBank RDRP references.

| GenBank ID | Host | Country code | Years |
|---|---|---|---|
| AB186898 | *NA THA* | NA | NA |
| AB517731 | *NA IND* | IND | 2007 |
| AB517732 | *NA IND* | IND | 2007 |
| AB517733 | *NA IND* | IND | 2007 |
| AB517734 | *NA IND* | IND | 2007 |
| AB517735 | *NA IND* | IND | 2007 |
| AB517736 | *NA IND* | IND | 2007 |
| AB517737 | *NA IND* | IND | 2007 |
| AB517738 | *NA IND* | IND | 2007 |
| AB517739 | *NA IND* | IND | 2008 |
| AB828072 | *Bos taurus* | CHN | NA |
| AF246939 | *NA* | TCD | NA |
| AF246940 | *NA* | NA | NA |
| GQ221268 | *Bos indicus* | IND | 2005 |
| GQ915026 | *Homo sapiens* | TCD | 2004 |
| GQ915029 | *Homo sapiens* | TCD | NA |
| GU968924 | *Homo sapiens* | NLD | 2007 |
| HM070240 | *Sus scrofa* | CHN | 2010 |
| JF755419 | *Mus musculus* | TCD | 2008 |
| JF755420 | *Microtus pennsylvani-cus* | TCD | 2008 |
| JQ710506 | *Macaca mulatta* | CHN | 2002 |
| JQ710507 | *Macaca mulatta* | CHN | 2002 |
| JQ776552 | *Zalophus californianus* | AGO | 2008 |
| KC692366 | *Vulpes vulpes* | NLD | 2012 |
| KF792838 | *Felis Felis catusus* | PRT | 2012 |
| KF823810 | *Vulpes vulpes* | ESP | 2013 |
| KF823811 | *Vulpes vulpes* | ESP | 2013 |
| KF861773 | *Sus scrofa* | ITA | 2004 |
| KJ206569 | *Homo sapiens* | NLD | NA |
| KJ495690 | *Meleagris gallopavo* | TCD | 2011 |
| KJ663814 | *Homo sapiens* | TCD | 2013 |
| KJ663816 | *Homo sapiens* | TCD | 2013 |
| KM254161 | *Gallus gallus* | ZAF | 2013 |
| KM254162 | *Gallus gallus* | ZAF | 2013 |
| KM254164 | *Gallus gallus* | ZAF | 2013 |
| KM285233 | *Homo sapiens* | KHM | 2009 |
| KM285234 | *Homo sapiens* | KHM | 2009 |
| KM573798 | *Camelus dromedarius* | ARE | 2013 |
| KM573799 | *Camelus dromedarius* | ARE | 2013 |
| KM573800 | *Camelus dromedarius* | ARE | 2013 |
| KM573801 | *Camelus dromedarius* | ARE | 2013 |
| KM573802 | *Camelus dromedarius* | ARE | 2013 |
| KM573803 | *Camelus dromedarius* | ARE | 2013 |
| KM573804 | *Camelus dromedarius* | ARE | 2013 |
| KM573805 | *Camelus dromedarius* | ARE | 2013 |
| KM573806 | *Camelus dromedarius* | ARE | 2013 |
| KM573807 | *Camelus dromedarius* | ARE | 2013 |
| KM573808 | *Camelus dromedarius* | ARE | 2013 |
| KM573809 | *Camelus dromedarius* | ARE | 2013 |
| KP941111 | *Vulpes vulpes* | HRV | 2014 |
| KP984805 | *Sus scrofa* | CHN | 2012 |
| KR106195 | *Arctocephalus australis* | BRA | 2012 |
| KR827412 | *Homo sapiens* | CHN | 2012 |
| KR827413 | *Homo sapiens* | CHN | 2012 |
| KR827414 | *Homo sapiens* | CHN | 2012 |

| | | | |
|---|---|---|---|
| KR827415 | *Homo sapiens* | CHN | 2013 |
| KR827416 | *Homo sapiens* | CHN | 2013 |
| KR827417 | *Homo sapiens* | CHN | 2014 |
| KR827418 | *Homo sapiens* | CHN | 2013 |
| KR902502 | *Equus caballus* | TCD | 2012 |
| KR902503 | *Equus caballus* | TCD | 2012 |
| KR902505 | *Equus caballus* | TCD | 2012 |
| KR902507 | *Equus caballus* | TCD | 2012 |
| KT934307 | *Canis lupus* | PRT | 2015 |
| KT934308 | *Canis lupus* | PRT | 2015 |
| KT984499 | *Macaca mulatta* | TCD | 2014 |
| KU729755 | *Zalophus californianus* | AGO | 2008 |
| KU729756 | *Zalophus californianus* | AGO | 2008 |
| KU729757 | *Zalophus californianus* | AGO | 2008 |
| KU729758 | *Zalophus californianus* | AGO | 2008 |
| KU729759 | *Zalophus californianus* | AGO | 2008 |
| KU729760 | *Zalophus californianus* | AGO | 2008 |
| KU729761 | *Zalophus californianus* | AGO | 2009 |
| KU729762 | *Zalophus californianus* | AGO | 2009 |
| KU729763 | *Zalophus californianus* | AGO | 2009 |
| KU729764 | *Zalophus californianus* | AGO | 2009 |
| KU729765 | *Zalophus californianus* | AGO | 2009 |
| KU729766 | *Zalophus californianus* | AGO | 2009 |
| KU729767 | *Zalophus californianus* | AGO | 2009 |
| KU729768 | *Zalophus californianus* | AGO | 2010 |
| KU729769 | *Zalophus californianus* | AGO | 2010 |
| KU892528 | *Homo sapiens* | BEL | 2010 |
| KU892529 | *Homo sapiens* | BEL | 2010 |
| KU892530 | *Homo sapiens* | BEL | 2010 |
| KX374476 | *Bos taurus* | IND | 2015 |
| KX374477 | *Sus scrofa* | IND | 2013 |
| KX374478 | *Sus scrofa* | IND | 2013 |
| KY053140 | *Chlorocebus sabaeus* | KNA | 2015 |
| KY053141 | *Chlorocebus sabaeus* | KNA | 2015 |
| KY053142 | *Chlorocebus sabaeus* | KNA | 2015 |
| KY053143 | *Chlorocebus sabaeus* | KNA | 2015 |
| KY120170 | *Felis catustle* | AGO | 2012 |
| KY120171 | *Felis catustle* | AGO | 2012 |
| KY120172 | *Felis catustle* | AGO | 2012 |
| KY120173 | *Felis catustle* | AGO | 2012 |
| KY120174 | *Felis catustle* | AGO | 2012 |
| KY120175 | *Felis catustle* | AGO | 2012 |
| KY120176 | *Felis catustle* | AGO | 2012 |
| KY120177 | *Felis catustle* | AGO | 2012 |
| KY120178 | *Felis catustle* | AGO | 2012 |
| KY120179 | *Felis catustle* | AGO | 2012 |
| KY120180 | *Felis catustle* | AGO | 2012 |
| KY120181 | *Felis catustle* | AGO | 2012 |
| KY120182 | *Simiiformes* | AGO | 2009 |
| KY120183 | *Simiiformes* | AGO | 2009 |
| KY120184 | *Simiiformes* | AGO | 2009 |
| KY120185 | *Simiiformes* | AGO | 2009 |
| KY120186 | *Simiiformes* | AGO | 2009 |
| KY120187 | *Simiiformes* | AGO | 2009 |
| KY120188 | *Simiiformes* | AGO | 2009 |
| KY120189 | *Simiiformes* | AGO | 2009 |
| KY120190 | *Simiiformes* | AGO | 2009 |
| KY120191 | *Simiiformes* | AGO | 2009 |
| KY120192 | *Simiiformes* | AGO | 2009 |

| | | | |
|---|---|---|---|
| KY120193 | *Simiiformes* | AGO | 2009 |
| KY120194 | *Simiiformes* | AGO | 2009 |
| KY174983 | *Macaca mulatta* | TCD | 2012 |
| KY214430 | *Sus scrofa* | BEL | 2015 |
| KY214431 | *Sus scrofa* | BEL | 2015 |
| KY214432 | *Sus scrofa* | BEL | 2015 |
| KY399057 | *Canis lupus* | KNA | 2015 |
| KY502850 | *Gorilla gorilla* | COD | 2015 |
| KY502851 | *Gorilla gorilla* | COD | 2015 |
| KY502852 | *Gorilla gorilla* | COD | 2015 |
| KY502853 | *Gorilla gorilla* | COD | 2015 |
| KY502854 | *Gorilla gorilla* | COD | 2015 |
| KY502855 | *Gorilla gorilla* | COD | 2015 |
| KY502856 | *Gorilla gorilla* | COD | 2015 |
| KY502862 | *Gorilla gorilla* | COD | 2015 |
| KY502863 | *Gorilla gorilla* | COD | 2015 |
| KY502864 | *Gorilla gorilla* | COD | 2015 |
| KY502865 | *Gorilla gorilla* | COD | 2015 |
| KY502866 | *Gorilla gorilla* | COD | 2015 |
| KY502867 | *Gorilla gorilla* | COD | 2015 |
| KY502868 | *Gorilla gorilla* | COD | 2015 |
| KY502869 | *Gorilla gorilla* | COD | 2015 |
| KY502870 | *Gorilla gorilla* | COD | 2015 |
| KY502871 | *Gorilla gorilla* | COD | 2015 |
| KY502872 | *Gorilla gorilla* | COD | 2015 |
| KY502873 | *Gorilla gorilla* | COD | 2015 |
| KY502874 | *Gorilla gorilla* | COD | 2015 |
| KY502875 | *Gorilla gorilla* | COD | 2015 |
| KY502876 | *Gorilla gorilla* | COD | 2015 |
| KY502879 | *Gorilla gorilla* | COD | 2015 |
| KY502961 | *Gorilla gorilla* | COD | 2015 |
| KY502962 | *Gorilla gorilla* | COD | 2015 |
| KY502963 | *Gorilla gorilla* | COD | 2015 |
| KY502964 | *Gorilla gorilla* | COD | 2015 |
| KY502965 | *Gorilla gorilla* | COD | 2015 |
| KY502966 | *Gorilla gorilla* | COD | 2015 |
| KY502967 | *Gorilla gorilla* | COD | 2015 |
| KY502968 | *Gorilla gorilla* | COD | 2015 |
| KY502969 | *Gorilla gorilla* | COD | 2015 |
| KY502970 | *Gorilla gorilla* | COD | 2015 |
| KY502978 | *Gorilla gorilla* | COD | 2015 |
| KY502979 | *Gorilla gorilla* | COD | 2015 |
| KY502980 | *Gorilla gorilla* | COD | 2015 |
| KY502982 | *Gorilla gorilla* | COD | 2015 |
| KY502985 | *Gorilla gorilla* | COD | 2015 |
| KY502986 | *Gorilla gorilla* | COD | 2015 |
| KY502987 | *Gorilla gorilla* | COD | 2015 |
| KY502988 | *Gorilla gorilla* | COD | 2015 |
| KY502989 | *Gorilla gorilla* | COD | 2015 |
| KY502990 | *Gorilla gorilla* | COD | 2015 |
| KY502991 | *Gorilla gorilla* | COD | 2015 |
| KY502992 | *Gorilla gorilla* | COD | 2015 |
| KY502993 | *Gorilla gorilla* | COD | 2015 |
| KY502994 | *Gorilla gorilla* | COD | 2015 |
| KY502996 | *Gorilla gorilla* | COD | 2015 |
| KY502998 | *Gorilla gorilla* | COD | 2015 |
| KY502999 | *Gorilla gorilla* | COD | 2015 |
| KY503001 | *Gorilla gorilla* | COD | 2015 |
| KY503004 | *Gorilla gorilla* | COD | 2015 |

| | | | |
|---|---|---|---|
| KY503005 | *Gorilla gorilla* | COD | 2015 |
| KY503009 | *Gorilla gorilla* | COD | 2015 |
| KY503010 | *Gorilla gorilla* | COD | 2015 |
| KY503020 | *Gorilla gorilla* | COD | 2015 |
| KY855428 | *Marmota himalayana* | CHN | 2013 |
| KY855429 | *Marmota himalayana* | CHN | 2013 |
| KY855430 | *Marmota himalayana* | CHN | 2013 |
| KY855431 | *Marmota himalayana* | CHN | 2013 |
| KY928683 | *Marmota himalayana* | CHN | 2013 |
| KY928684 | *Marmota himalayana* | CHN | 2013 |
| KY928685 | *Marmota himalayana* | CHN | 2013 |
| KY928686 | *Marmota himalayana* | CHN | 2013 |
| KY928687 | *Marmota himalayana* | CHN | 2013 |
| KY928688 | *Marmota himalayana* | CHN | 2013 |
| KY928689 | *Marmota himalayana* | CHN | 2013 |
| KY928690 | *Marmota himalayana* | CHN | 2013 |
| KY928691 | *Marmota himalayana* | CHN | 2013 |
| KY928692 | *Marmota himalayana* | CHN | 2013 |
| KY928693 | *Marmota himalayana* | CHN | 2013 |
| KY928694 | *Marmota himalayana* | CHN | 2013 |
| KY928695 | *Marmota himalayana* | CHN | 2013 |
| KY928696 | *Marmota himalayana* | CHN | 2013 |
| KY928697 | *Marmota himalayana* | CHN | 2013 |
| KY928698 | *Marmota himalayana* | CHN | 2013 |
| KY928699 | *Marmota himalayana* | CHN | 2013 |
| KY928700 | *Marmota himalayana* | CHN | 2013 |
| KY928701 | *Marmota himalayana* | CHN | 2013 |
| KY928702 | *Marmota himalayana* | CHN | 2013 |
| KY928703 | *Marmota himalayana* | CHN | 2013 |
| KY928704 | *Marmota himalayana* | CHN | 2013 |
| KY928705 | *Marmota himalayana* | CHN | 2013 |
| KY928706 | *Marmota himalayana* | CHN | 2013 |
| KY928707 | *Marmota himalayana* | CHN | 2013 |
| KY928708 | *Marmota himalayana* | CHN | 2013 |
| KY928709 | *Marmota himalayana* | CHN | 2013 |
| KY928710 | *Marmota himalayana* | CHN | 2013 |
| KY928711 | *Marmota himalayana* | CHN | 2013 |
| KY928712 | *Marmota himalayana* | CHN | 2013 |
| KY928713 | *Marmota himalayana* | CHN | 2013 |
| KY928714 | *Marmota himalayana* | CHN | 2013 |
| KY928715 | *Marmota himalayana* | CHN | 2013 |
| KY928716 | *Marmota himalayana* | CHN | 2013 |
| KY928717 | *Marmota himalayana* | CHN | 2013 |
| KY928718 | *Marmota himalayana* | CHN | 2013 |
| KY928719 | *Marmota himalayana* | CHN | 2013 |
| KY928720 | *Marmota himalayana* | CHN | 2013 |
| KY928721 | *Marmota himalayana* | CHN | 2013 |
| KY928722 | *Marmota himalayana* | CHN | 2013 |
| KY928723 | *Marmota himalayana* | CHN | 2013 |
| KY928724 | *Marmota himalayana* | CHN | 2013 |
| KY928725 | *Marmota himalayana* | CHN | 2013 |
| KY928726 | *Marmota himalayana* | CHN | 2013 |
| KY928727 | *Marmota himalayana* | CHN | 2013 |
| KY928728 | *Marmota himalayana* | CHN | 2013 |
| KY928729 | *Marmota himalayana* | CHN | 2013 |
| KY928730 | *Marmota himalayana* | CHN | 2013 |
| KY928731 | *Marmota himalayana* | CHN | 2013 |
| KY928732 | *Marmota himalayana* | CHN | 2013 |
| KY928733 | *Marmota himalayana* | CHN | 2013 |

| | | | |
|---|---|---|---|
| KY928734 | *Marmota himalayana* | CHN | 2013 |
| KY928735 | *Marmota himalayana* | CHN | 2013 |
| KY928736 | *Marmota himalayana* | CHN | 2013 |
| KY928737 | *Marmota himalayana* | CHN | 2013 |
| KY928738 | *Marmota himalayana* | CHN | 2013 |
| LC110353 | *Mus musculus* | JPN | 2015 |
| LC338002 | *Camelus dromedaries* | ARE | 2013 |
| LC338003 | *Camelus dromedaries* | ARE | 2013 |
| LC338004 | *Camelus dromedaries* | ARE | 2013 |
| LC338005 | *Camelus dromedaries* | ARE | 2013 |
| LC338006 | *Camelus dromedaries* | ARE | 2013 |
| LC338007 | *Camelus dromedaries* | ARE | 2013 |
| LC338008 | *Camelus dromedaries* | ARE | 2013 |
| LC338009 | *Camelus dromedaries* | ARE | 2013 |
| MF071281 | *Felis catus* | KNA | 2014 |
| MF416389 | *Mus musculus* | TCD | 2015 |
| MF416390 | *Mus musculus* | TCD | 2014 |
| MF416391 | *Mus musculus* | TCD | 2014 |
| MG003334 | *Camelus dromedarius* | IND | 2016 |
| MG003339 | *Bos taurus* | IND | 2015 |
| MG003340 | *Bos taurus* | IND | 2015 |
| MG003341 | *Bos taurus* | IND | 2015 |
| MG010904 | *Macaca mulatta* | TCD | 2011 |
| MG010905 | *Macaca mulatta* | TCD | 2011 |
| MG010906 | *Macaca mulatta* | TCD | 2011 |
| MG010907 | *Macaca mulatta* | TCD | 2011 |
| MG010908 | *Macaca mulatta* | TCD | 2011 |
| MG010909 | *Macaca mulatta* | TCD | 2011 |
| MG010910 | *Macaca mulatta* | TCD | 2011 |
| MG010911 | *Macaca mulatta* | TCD | 2011 |
| MG010912 | *Macaca mulatta* | TCD | 2011 |
| MG010913 | *Macaca mulatta* | TCD | 2011 |
| MG010915 | *Macaca mulatta* | TCD | 2011 |
| MG010916 | *Macaca mulatta* | TCD | 2011 |
| MG010917 | *Macaca mulatta* | TCD | 2011 |
| MG010918 | *Macaca mulatta* | TCD | 2011 |
| MG010919 | *Macaca mulatta* | TCD | 2011 |
| MG010920 | *Macaca mulatta* | TCD | 2011 |
| MG010921 | *Macaca mulatta* | TCD | 2011 |
| MG190029 | *Roe deer* | SVN | 2014 |
| MG571903 | *Homo sapiens* | SEN | 2015 |
| MG571907 | *Homo sapiens* | SEN | 2015 |
| MG600063 | *Parupeneus cyclostomus* | CHN | NA |
| MG600064 | *Tropidophorus sinicus* | CHN | NA |
| MG821233 | *Caprine* | IND | 2015 |
| MG846401 | *Gallus gallus* | BRA | 2015 |
| MG846402 | *Gallus gallus* | BRA | 2015 |
| MG846403 | *Gallus gallus* | BRA | 2015 |
| MG846404 | *Gallus gallus* | BRA | 2015 |
| MG846405 | *Gallus gallus* | BRA | 2015 |
| MG846406 | *Gallus gallus* | BRA | 2015 |
| MG846407 | *Gallus gallus* | BRA | 2015 |
| MG846408 | *Gallus gallus* | BRA | 2015 |
| MG846409 | *Gallus gallus* | BRA | 2015 |
| MG846410 | *Gallus gallus* | BRA | 2015 |
| MG846411 | *Gallus gallus* | BRA | 2015 |
| MG846412 | *Gallus gallus* | BRA | 2015 |
| MH327934 | *Gallus gallus* | HUN | 2011 |

| | | | |
|---|---|---|---|
| MH412924 | *Rattus sp.* | KNA | 2017 |
| MH453875 | *Australian Shelduck* | AUS | 2012 |
| MH453878 | *Australian Shelduck* | AUS | 2012 |
| MH933801 | *Homo sapiens* | CMR | 2014 |
| MH933802 | *Homo sapiens* | CMR | 2014 |
| MH933803 | *Homo sapiens* | CMR | 2014 |
| MH933804 | *Homo sapiens* | CMR | 2014 |
| MH933805 | *Homo sapiens* | CMR | 2014 |
| MH933806 | *Homo sapiens* | CMR | 2014 |
| MH933807 | *Homo sapiens* | CMR | 2014 |
| MH933808 | *Homo sapiens* | CMR | 2014 |
| MH933809 | *Homo sapiens* | CMR | 2014 |
| MH933810 | *Homo sapiens* | CMR | 2014 |
| MH933811 | *Homo sapiens* | CMR | 2014 |
| MH933812 | *Homo sapiens* | CMR | 2014 |
| MH933813 | *Homo sapiens* | CMR | 2014 |
| MH933814 | *Homo sapiens* | CMR | 2014 |
| MH933815 | *Homo sapiens* | CMR | 2014 |
| MH933817 | *Homo sapiens* | CMR | 2014 |
| MH933818 | *Homo sapiens* | CMR | 2014 |
| MH933819 | *Homo sapiens* | CMR | 2014 |
| MH933820 | *Homo sapiens* | CMR | 2014 |
| MH933821 | *Homo sapiens* | CMR | 2014 |
| MH933822 | *Homo sapiens* | CMR | 2014 |
| MH933823 | *Homo sapiens* | CMR | 2014 |
| MH933824 | *Homo sapiens* | CMR | 2014 |
| MH933825 | *Homo sapiens* | CMR | 2014 |
| MH933830 | *Homo sapiens* | CMR | 2014 |
| MH933831 | *Homo sapiens* | CMR | 2014 |
| MH933832 | *Homo sapiens* | CMR | 2014 |
| MH933833 | *Homo sapiens* | CMR | 2014 |
| MH933834 | *Homo sapiens* | CMR | 2014 |
| MH933835 | *Homo sapiens* | CMR | 2014 |
| MH933836 | *Homo sapiens* | CMR | 2014 |
| MH933839 | *Homo sapiens* | CMR | 2014 |
| MH933841 | *Homo sapiens* | CMR | 2014 |
| MK064212 | *Chiroptera* | CHN | 2016 |
| MK064213 | *Chiroptera* | CHN | 2016 |
| MK204395 | *Anas gracilis* | AUS | 2017 |
| MK204418 | *Malacorhynchus mem-branaceus* | AUS | 2017 |
| MK305310 | *Homo sapiens* | AGO | 2018 |
| MK378834 | *Sus scrofa* | CHN | 2017 |
| MK378835 | *Sus scrofa* | CHN | 2017 |
| MK378843 | *Sus scrofa* | CHN | 2017 |
| MK378844 | *Sus scrofa* | CHN | 2017 |
| MK378845 | *Sus scrofa* | CHN | 2017 |
| MK378851 | *Sus scrofa* | CHN | 2017 |
| MK378856 | *Sus scrofa* | CHN | 2017 |
| MK378859 | *Sus scrofa* | CHN | 2017 |
| MK378860 | *Sus scrofa* | CHN | 2017 |
| MK378865 | *Sus scrofa* | CHN | 2017 |
| MK378866 | *Sus scrofa* | CHN | 2017 |
| MK378867 | *Sus scrofa* | CHN | 2017 |
| MK378868 | *Sus scrofa* | CHN | 2017 |
| MK378869 | *Sus scrofa* | CHN | 2017 |
| MK378870 | *Sus scrofa* | CHN | 2017 |
| MK378876 | *Sus scrofa* | CHN | 2017 |
| MK521919 | *Sarcophilus harrisii* | AUS | 2017 |

| | | | |
|---|---|---|---|
| MK521920 | *Sarcophilus harrisii* | AUS | 2016 |
| MK521921 | *Sarcophilus harrisii* | AUS | 2017 |
| MK521922 | *Sarcophilus harrisii* | AUS | 2017 |
| MK521923 | *Sarcophilus harrisii* | AUS | 2017 |
| MK521924 | *Sarcophilus harrisii* | AUS | 2016 |
| MK521925 | *Sarcophilus harrisii* | AUS | 2016 |
| MK521926 | *Sarcophilus harrisii* | AUS | 2017 |
| MN145873 | *Homo sapiens* | CHN | 2018 |
| MN563295 | *Urva auropunctata* | KNA | 2017 |
| MN563296 | *Urva auropunctata* | KNA | 2017 |
| MN563297 | *Urva auropunctata* | KNA | 2017 |
| MN563298 | *Urva auropunctata* | KNA | 2017 |
| MN563299 | *Urva auropunctata* | KNA | 2017 |
| MN563300 | *Urva auropunctata* | KNA | 2017 |
| MN563301 | *Urva auropunctata* | KNA | 2017 |
| MN563302 | *Urva auropunctata* | KNA | 2017 |
| MN692671 | *Macaca fascicularis* | THA | 2017 |
| MN871976 | *Chlorocebus sabaeus* | CHN | 2018 |
| MT129742 | *NA* | AUS | 2017 |
| MT129743 | *NA* | AUS | 2017 |
| MT129744 | *NA* | AUS | 2017 |
| MT129745 | *NA* | AUS | 2017 |
| MT129746 | *NA* | AUS | 2017 |
| MT129747 | *NA* | AUS | 2017 |
| MT129748 | *NA* | AUS | 2017 |
| MT129749 | *NA* | AUS | 2017 |
| MT129750 | *NA* | AUS | 2017 |
| MT129751 | *NA* | AUS | 2017 |
| MT129752 | *NA* | AUS | 2017 |
| MT129753 | *NA* | AUS | 2017 |
| MT150089 | *NA* | NA | 2018 |
| MT341487 | *Actinonaias pectorosa* | TCD | 2018 |
| MT350351 | *Pan troglodytes* | SLE | NA |
| MT350352 | *Pan troglodytes* | SLE | NA |
| MT846991 | *Pantholops hodgsonii* | CHN | 2014 |
| MT847000 | *Pantholops hodgsonii* | CHN | 2014 |
| MT847001 | *Pantholops hodgsonii* | CHN | 2014 |
| MT847002 | *Pantholops hodgsonii* | CHN | 2014 |
| MT847003 | *Pantholops hodgsonii* | CHN | 2014 |
| MT847004 | *Pantholops hodgsonii* | CHN | 2014 |
| MT847005 | *Pantholops hodgsonii* | CHN | 2014 |
| MT847006 | *Pantholops hodgsonii* | CHN | 2014 |
| MT847007 | *Pantholops hodgsonii* | CHN | 2014 |
| MT847008 | *Pantholops hodgsonii* | CHN | 2014 |
| MT847009 | *Pantholops hodgsonii* | CHN | 2014 |
| MT847010 | *Pantholops hodgsonii* | CHN | 2014 |
| MT847011 | *Pantholops hodgsonii* | CHN | 2014 |
| MT847012 | *Pantholops hodgsonii* | CHN | 2014 |
| NC_007027 | *NA* | THA | NA |
| NC_29802 | *Sus scrofa* | ITA | 2004 |
| NC_34161 | *Zalophus californianus* | AGO | 2008 |
| NC_34452 | *Chlorocebus sabaeus* | KNA | 2015 |
| NC_35206 | *Canis lupus* | KNA | 2015 |
| NC_40439 | *Gallus gallus* | HUN | 2011 |
| NC_40753 | *Roe deer* | SVN | 2014 |

**Supplemental Table S2.** Listing of GenBank Capsid references.

| GenBank ID | Host | Country code | Years |
|---|---|---|---|
| JQ776551 | *Zalophus californianus* | AGO | 2005 |
| KF861768 | *Sus scrofa* | ITA | 2004 |
| KF861770 | *Sus scrofa* | ITA | 2004 |
| KF861771 | *Sus scrofa* | ITA | 2004 |
| KF861772 | *Sus scrofa* | ITA | 2004 |
| KJ206568 | *Homo sapiens* | NLD | NA |
| KJ495689 | *Meleagris gallopavo* | TCD | 2011 |
| KJ663815 | *Homo sapiens* | TCD | 2013 |
| KM573778 | *Camelus dromedarius* | ARE | 2013 |
| KM573779 | *Camelus dromedarius* | ARE | 2013 |
| KM573780 | *Camelus dromedarius* | ARE | 2013 |
| KM573781 | *Camelus dromedarius* | ARE | 2013 |
| KM573782 | *Camelus dromedarius* | ARE | 2013 |
| KM573783 | *Camelus dromedarius* | ARE | 2013 |
| KM573784 | *Camelus dromedarius* | ARE | 2013 |
| KM573785 | *Camelus dromedarius* | ARE | 2013 |
| KM573786 | *Camelus dromedarius* | ARE | 2013 |
| KM573787 | *Camelus dromedarius* | ARE | 2013 |
| KM573788 | *Camelus dromedarius* | ARE | 2013 |
| KM573789 | *Camelus dromedarius* | ARE | 2013 |
| KM573790 | *Camelus dromedarius* | ARE | 2013 |
| KM573791 | *Camelus dromedarius* | ARE | 2013 |
| KM573792 | *Camelus dromedarius* | ARE | 2013 |
| KM573794 | *Camelus dromedarius* | ARE | 2013 |
| KM573795 | *Camelus dromedarius* | ARE | 2013 |
| KM573796 | *Camelus dromedarius* | ARE | 2013 |
| KM573797 | *Camelus dromedarius* | ARE | 2013 |
| KR902502 | *Equus caballus* | TCD | 2012 |
| KR902504 | *Equus caballus* | TCD | 2012 |
| KR902506 | *Equus caballus* | TCD | 2012 |
| KR902508 | *Equus caballus* | TCD | 2012 |
| KT934309 | *Canis lupus* | PRT | 2015 |
| KT934310 | *Canis lupus* | PRT | 2015 |
| KU729746 | *Zalophus californianus* | AGO | 2008 |
| KU729747 | *Zalophus californianus* | AGO | 2009 |
| KU729748 | *Zalophus californianus* | AGO | 2009 |
| KU729749 | *Zalophus californianus* | AGO | 2008 |
| KU729750 | *Zalophus californianus* | AGO | 2009 |
| KU729751 | *Zalophus californianus* | AGO | 2009 |
| KU729752 | *Zalophus californianus* | AGO | 2009 |
| KU729753 | *Zalophus californianus* | AGO | 2009 |
| KU729754 | *Zalophus californianus* | AGO | 2008 |
| KU892524 | *Homo sapiens* | BEL | 2010 |
| KU892525 | *Homo sapiens* | BEL | 2010 |
| KU892526 | *Homo sapiens* | BEL | 2010 |
| KU892527 | *Homo sapiens* | BEL | 2010 |
| KY174982 | *Macaca mulatta* | TCD | 2012 |
| KY214426 | *Sus scrofa* | BEL | 2015 |
| KY214427 | *Sus scrofa* | BEL | 2015 |
| KY214428 | *Sus scrofa* | BEL | 2015 |
| KY214429 | *Sus scrofa* | BEL | 2015 |
| KY502835 | *Gorilla gorilla* | COD | 2015 |
| KY502836 | *Gorilla gorilla* | COD | 2015 |
| KY502837 | *Gorilla gorilla* | COD | 2015 |
| KY502838 | *Gorilla gorilla* | COD | 2015 |
| KY502839 | *Gorilla gorilla* | COD | 2015 |

| | | | |
|---|---|---|---|
| KY502840 | *Gorilla gorilla* | COD | 2015 |
| KY502841 | *Gorilla gorilla* | COD | 2015 |
| KY502842 | *Gorilla gorilla* | COD | 2015 |
| KY502843 | *Gorilla gorilla* | COD | 2015 |
| KY502845 | *Gorilla gorilla* | COD | 2015 |
| KY502846 | *Gorilla gorilla* | COD | 2015 |
| KY502847 | *Gorilla gorilla* | COD | 2015 |
| KY502848 | *Gorilla gorilla* | COD | 2015 |
| KY502849 | *Gorilla gorilla* | COD | 2015 |
| KY502857 | *Gorilla gorilla* | COD | 2015 |
| KY502858 | *Gorilla gorilla* | COD | 2015 |
| KY502859 | *Gorilla gorilla* | COD | 2015 |
| KY502860 | *Gorilla gorilla* | COD | 2015 |
| KY502878 | *Gorilla gorilla* | COD | 2015 |
| KY502932 | *Gorilla gorilla* | COD | 2015 |
| KY502935 | *Gorilla gorilla* | COD | 2015 |
| KY502937 | *Gorilla gorilla* | COD | 2015 |
| KY502943 | *Gorilla gorilla* | COD | 2015 |
| KY502944 | *Gorilla gorilla* | COD | 2015 |
| KY502946 | *Gorilla gorilla* | COD | 2015 |
| KY502947 | *Gorilla gorilla* | COD | 2015 |
| KY502948 | *Gorilla gorilla* | COD | 2015 |
| KY502951 | *Gorilla gorilla* | COD | 2015 |
| KY502952 | *Gorilla gorilla* | COD | 2015 |
| KY502953 | *Gorilla gorilla* | COD | 2015 |
| KY502972 | *Gorilla gorilla* | COD | 2015 |
| KY502973 | *Gorilla gorilla* | COD | 2015 |
| KY502975 | *Gorilla gorilla* | COD | 2015 |
| KY502977 | *Gorilla gorilla* | COD | 2015 |
| KY503014 | *Gorilla gorilla* | COD | 2015 |
| KY855428 | *Marmota himalayana* | CHN | 2013 |
| KY855429 | *Marmota himalayana* | CHN | 2013 |
| KY855430 | *Marmota himalayana* | CHN | 2013 |
| KY855431 | *Marmota himalayana* | CHN | 2013 |
| KY928739 | *Marmota himalayana* | CHN | 2013 |
| KY928740 | *Marmota himalayana* | CHN | 2013 |
| KY928741 | *Marmota himalayana* | CHN | 2013 |
| KY928742 | *Marmota himalayana* | CHN | 2013 |
| KY928743 | *Marmota himalayana* | CHN | 2013 |
| KY928744 | *Marmota himalayana* | CHN | 2013 |
| KY928745 | *Marmota himalayana* | CHN | 2013 |
| KY928746 | *Marmota himalayana* | CHN | 2013 |
| KY928747 | *Marmota himalayana* | CHN | 2013 |
| KY928748 | *Marmota himalayana* | CHN | 2013 |
| KY928749 | *Marmota himalayana* | CHN | 2013 |
| KY928750 | *Marmota himalayana* | CHN | 2013 |
| KY928751 | *Marmota himalayana* | CHN | 2013 |
| KY928752 | *Marmota himalayana* | CHN | 2013 |
| KY928753 | *Marmota himalayana* | CHN | 2013 |
| KY928754 | *Marmota himalayana* | CHN | 2013 |
| KY928755 | *Marmota himalayana* | CHN | 2013 |
| KY928756 | *Marmota himalayana* | CHN | 2013 |
| KY928757 | *Marmota himalayana* | CHN | 2013 |
| KY928758 | *Marmota himalayana* | CHN | 2013 |
| KY928759 | *Marmota himalayana* | CHN | 2013 |
| KY928760 | *Marmota himalayana* | CHN | 2013 |
| KY928761 | *Marmota himalayana* | CHN | 2013 |
| KY928762 | *Marmota himalayana* | CHN | 2013 |
| KY928763 | *Marmota himalayana* | CHN | 2013 |

| | | | |
|---|---|---|---|
| KY928764 | *Marmota himalayana* | CHN | 2013 |
| KY928765 | *Marmota himalayana* | CHN | 2013 |
| KY928766 | *Marmota himalayana* | CHN | 2013 |
| KY928767 | *Marmota himalayana* | CHN | 2013 |
| KY928768 | *Marmota himalayana* | CHN | 2013 |
| KY928769 | *Marmota himalayana* | CHN | 2013 |
| KY928770 | *Marmota himalayana* | CHN | 2013 |
| KY928771 | *Marmota himalayana* | CHN | 2013 |
| KY928772 | *Marmota himalayana* | CHN | 2013 |
| KY928773 | *Marmota himalayana* | CHN | 2013 |
| KY928774 | *Marmota himalayana* | CHN | 2013 |
| KY928775 | *Marmota himalayana* | CHN | 2013 |
| KY928776 | *Marmota himalayana* | CHN | 2013 |
| KY928777 | *Marmota himalayana* | CHN | 2013 |
| KY928778 | *Marmota himalayana* | CHN | 2013 |
| KY928779 | *Marmota himalayana* | CHN | 2013 |
| KY928780 | *Marmota himalayana* | CHN | 2013 |
| KY928781 | *Marmota himalayana* | CHN | 2013 |
| KY928782 | *Marmota himalayana* | CHN | 2013 |
| KY928783 | *Marmota himalayana* | CHN | 2013 |
| KY928784 | *Marmota himalayana* | CHN | 2013 |
| KY928785 | *Marmota himalayana* | CHN | 2013 |
| KY928786 | *Marmota himalayana* | CHN | 2013 |
| KY928787 | *Marmota himalayana* | CHN | 2013 |
| KY928788 | *Marmota himalayana* | CHN | 2013 |
| KY928789 | *Marmota himalayana* | CHN | 2013 |
| KY928790 | *Marmota himalayana* | CHN | 2013 |
| KY928791 | *Marmota himalayana* | CHN | 2013 |
| KY928792 | *Marmota himalayana* | CHN | 2013 |
| KY928793 | *Marmota himalayana* | CHN | 2013 |
| KY928794 | *Marmota himalayana* | CHN | 2013 |
| KY928795 | *Marmota himalayana* | CHN | 2013 |
| KY928796 | *Marmota himalayana* | CHN | 2013 |
| KY928797 | *Marmota himalayana* | CHN | 2013 |
| KY928798 | *Marmota himalayana* | CHN | 2013 |
| KY928799 | *Marmota himalayana* | CHN | 2013 |
| KY928800 | *Marmota himalayana* | CHN | 2013 |
| KY928801 | *Marmota himalayana* | CHN | 2013 |
| KY928802 | *Marmota himalayana* | CHN | 2013 |
| KY928803 | *Marmota himalayana* | CHN | 2013 |
| KY928804 | *Marmota himalayana* | CHN | 2013 |
| KY928805 | *Marmota himalayana* | CHN | 2013 |
| KY928806 | *Marmota himalayana* | CHN | 2013 |
| KY928807 | *Marmota himalayana* | CHN | 2013 |
| KY928808 | *Marmota himalayana* | CHN | 2013 |
| KY928809 | *Marmota himalayana* | CHN | 2013 |
| KY928810 | *Marmota himalayana* | CHN | 2013 |
| KY928811 | *Marmota himalayana* | CHN | 2013 |
| KY928812 | *Marmota himalayana* | CHN | 2013 |
| KY928813 | *Marmota himalayana* | CHN | 2013 |
| KY928814 | *Marmota himalayana* | CHN | 2013 |
| KY928815 | *Marmota himalayana* | CHN | 2013 |
| KY928816 | *Marmota himalayana* | CHN | 2013 |
| KY928817 | *Marmota himalayana* | CHN | 2013 |
| KY928818 | *Marmota himalayana* | CHN | 2013 |
| KY928819 | *Marmota himalayana* | CHN | 2013 |
| KY928820 | *Marmota himalayana* | CHN | 2013 |
| KY928821 | *Marmota himalayana* | CHN | 2013 |
| KY928822 | *Marmota himalayana* | CHN | 2013 |

| | | | |
|---|---|---|---|
| KY928823 | *Marmota himalayana* | CHN | 2013 |
| KY928824 | *Marmota himalayana* | CHN | 2013 |
| KY928825 | *Marmota himalayana* | CHN | 2013 |
| KY928826 | *Marmota himalayana* | CHN | 2013 |
| KY928827 | *Marmota himalayana* | CHN | 2013 |
| KY928828 | *Marmota himalayana* | CHN | 2013 |
| KY928829 | *Marmota himalayana* | CHN | 2013 |
| KY928830 | *Marmota himalayana* | CHN | 2013 |
| KY928831 | *Marmota himalayana* | CHN | 2013 |
| KY928832 | *Marmota himalayana* | CHN | 2013 |
| KY928833 | *Marmota himalayana* | CHN | 2013 |
| KY928834 | *Marmota himalayana* | CHN | 2013 |
| KY928835 | *Marmota himalayana* | CHN | 2013 |
| KY928836 | *Marmota himalayana* | CHN | 2013 |
| KY928837 | *Marmota himalayana* | CHN | 2013 |
| KY928838 | *Marmota himalayana* | CHN | 2013 |
| KY928839 | *Marmota himalayana* | CHN | 2013 |
| KY928840 | *Marmota himalayana* | CHN | 2013 |
| KY928841 | *Marmota himalayana* | CHN | 2013 |
| KY928842 | *Marmota himalayana* | CHN | 2013 |
| KY928843 | *Marmota himalayana* | CHN | 2013 |
| KY928844 | *Marmota himalayana* | CHN | 2013 |
| KY928845 | *Marmota himalayana* | CHN | 2013 |
| KY928846 | *Marmota himalayana* | CHN | 2013 |
| KY928848 | *Marmota himalayana* | CHN | 2013 |
| KY928849 | *Marmota himalayana* | CHN | 2013 |
| KY928850 | *Marmota himalayana* | CHN | 2013 |
| KY928851 | *Marmota himalayana* | CHN | 2013 |
| KY928852 | *Marmota himalayana* | CHN | 2013 |
| KY928853 | *Marmota himalayana* | CHN | 2013 |
| KY928854 | *Marmota himalayana* | CHN | 2013 |
| KY928855 | *Marmota himalayana* | CHN | 2013 |
| KY928856 | *Marmota himalayana* | CHN | 2013 |
| KY928857 | *Marmota himalayana* | CHN | 2013 |
| KY928858 | *Marmota himalayana* | CHN | 2013 |
| KY928859 | *Marmota himalayana* | CHN | 2013 |
| KY928860 | *Marmota himalayana* | CHN | 2013 |
| KY928861 | *Marmota himalayana* | CHN | 2013 |
| KY928862 | *Marmota himalayana* | CHN | 2013 |
| KY928863 | *Marmota himalayana* | CHN | 2013 |
| KY928864 | *Marmota himalayana* | CHN | 2013 |
| KY928865 | *Marmota himalayana* | CHN | 2013 |
| KY928866 | *Marmota himalayana* | CHN | 2013 |
| KY928867 | *Marmota himalayana* | CHN | 2013 |
| KY928868 | *Marmota himalayana* | CHN | 2013 |
| KY928869 | *Marmota himalayana* | CHN | 2013 |
| KY928870 | *Marmota himalayana* | CHN | 2013 |
| KY928871 | *Marmota himalayana* | CHN | 2013 |
| KY928872 | *Marmota himalayana* | CHN | 2013 |
| KY928873 | *Marmota himalayana* | CHN | 2013 |
| KY928874 | *Marmota himalayana* | CHN | 2013 |
| KY928875 | *Marmota himalayana* | CHN | 2013 |
| KY928876 | *Marmota himalayana* | CHN | 2013 |
| KY928877 | *Marmota himalayana* | CHN | 2013 |
| KY928878 | *Marmota himalayana* | CHN | 2013 |
| KY928879 | *Marmota himalayana* | CHN | 2013 |
| KY928880 | *Marmota himalayana* | CHN | 2013 |
| KY928881 | *Marmota himalayana* | CHN | 2013 |
| KY928882 | *Marmota himalayana* | CHN | 2013 |

| | | | |
|---|---|---|---|
| KY928883 | *Marmota himalayana* | CHN | 2013 |
| KY928884 | *Marmota himalayana* | CHN | 2013 |
| KY928885 | *Marmota himalayana* | CHN | 2013 |
| KY928886 | *Marmota himalayana* | CHN | 2013 |
| KY928887 | *Marmota himalayana* | CHN | 2013 |
| KY928888 | *Marmota himalayana* | CHN | 2013 |
| KY928889 | *Marmota himalayana* | CHN | 2013 |
| KY928890 | *Marmota himalayana* | CHN | 2013 |
| KY928891 | *Marmota himalayana* | CHN | 2013 |
| KY928892 | *Marmota himalayana* | CHN | 2013 |
| KY928893 | *Marmota himalayana* | CHN | 2013 |
| KY928894 | *Marmota himalayana* | CHN | 2013 |
| KY928895 | *Marmota himalayana* | CHN | 2013 |
| KY928896 | *Marmota himalayana* | CHN | 2013 |
| KY928897 | *Marmota himalayana* | CHN | 2013 |
| KY928898 | *Marmota himalayana* | CHN | 2013 |
| KY928899 | *Marmota himalayana* | CHN | 2013 |
| KY928900 | *Marmota himalayana* | CHN | 2013 |
| KY928901 | *Marmota himalayana* | CHN | 2013 |
| KY928902 | *Marmota himalayana* | CHN | 2013 |
| KY928903 | *Marmota himalayana* | CHN | 2013 |
| KY928904 | *Marmota himalayana* | CHN | 2013 |
| KY928905 | *Marmota himalayana* | CHN | 2013 |
| KY928906 | *Marmota himalayana* | CHN | 2013 |
| KY928907 | *Marmota himalayana* | CHN | 2013 |
| KY928908 | *Marmota himalayana* | CHN | 2013 |
| KY928909 | *Marmota himalayana* | CHN | 2013 |
| KY928910 | *Marmota himalayana* | CHN | 2013 |
| KY928911 | *Marmota himalayana* | CHN | 2013 |
| KY928912 | *Marmota himalayana* | CHN | 2013 |
| KY928913 | *Marmota himalayana* | CHN | 2013 |
| KY928914 | *Marmota himalayana* | CHN | 2013 |
| KY928915 | *Marmota himalayana* | CHN | 2013 |
| KY928916 | *Marmota himalayana* | CHN | 2013 |
| KY928917 | *Marmota himalayana* | CHN | 2013 |
| KY928918 | *Marmota himalayana* | CHN | 2013 |
| KY928919 | *Marmota himalayana* | CHN | 2013 |
| KY928920 | *Marmota himalayana* | CHN | 2013 |
| KY928921 | *Marmota himalayana* | CHN | 2013 |
| KY928922 | *Marmota himalayana* | CHN | 2013 |
| KY928923 | *Marmota himalayana* | CHN | 2013 |
| KY928924 | *Marmota himalayana* | CHN | 2013 |
| KY928925 | *Marmota himalayana* | CHN | 2013 |
| KY928926 | *Marmota himalayana* | CHN | 2013 |
| KY928927 | *Marmota himalayana* | CHN | 2013 |
| KY928928 | *Marmota himalayana* | CHN | 2013 |
| KY928929 | *Marmota himalayana* | CHN | 2013 |
| KY928930 | *Marmota himalayana* | CHN | 2013 |
| KY928931 | *Marmota himalayana* | CHN | 2013 |
| KY928932 | *Marmota himalayana* | CHN | 2013 |
| KY928933 | *Marmota himalayana* | CHN | 2013 |
| KY928934 | *Marmota himalayana* | CHN | 2013 |
| KY928935 | *Marmota himalayana* | CHN | 2013 |
| KY928936 | *Marmota himalayana* | CHN | 2013 |
| KY928937 | *Marmota himalayana* | CHN | 2013 |
| KY928938 | *Marmota himalayana* | CHN | 2013 |
| KY928939 | *Marmota himalayana* | CHN | 2013 |
| KY928940 | *Marmota himalayana* | CHN | 2013 |
| KY928941 | *Marmota himalayana* | CHN | 2013 |

| | | | |
|---|---|---|---|
| KY928942 | *Marmota himalayana* | CHN | 2013 |
| KY928943 | *Marmota himalayana* | CHN | 2013 |
| KY928944 | *Marmota himalayana* | CHN | 2013 |
| KY928945 | *Marmota himalayana* | CHN | 2013 |
| KY928946 | *Marmota himalayana* | CHN | 2013 |
| KY928947 | *Marmota himalayana* | CHN | 2013 |
| KY928948 | *Marmota himalayana* | CHN | 2013 |
| KY928949 | *Marmota himalayana* | CHN | 2013 |
| KY928950 | *Marmota himalayana* | CHN | 2013 |
| KY928951 | *Marmota himalayana* | CHN | 2013 |
| KY928952 | *Marmota himalayana* | CHN | 2013 |
| KY928953 | *Marmota himalayana* | CHN | 2013 |
| KY928954 | *Marmota himalayana* | CHN | 2013 |
| KY928955 | *Marmota himalayana* | CHN | 2013 |
| KY928956 | *Marmota himalayana* | CHN | 2013 |
| KY928957 | *Marmota himalayana* | CHN | 2013 |
| KY928958 | *Marmota himalayana* | CHN | 2013 |
| KY928959 | *Marmota himalayana* | CHN | 2013 |
| KY928960 | *Marmota himalayana* | CHN | 2013 |
| KY928961 | *Marmota himalayana* | CHN | 2013 |
| KY928962 | *Marmota himalayana* | CHN | 2013 |
| KY928963 | *Marmota himalayana* | CHN | 2013 |
| KY928964 | *Marmota himalayana* | CHN | 2013 |
| KY928965 | *Marmota himalayana* | CHN | 2013 |
| KY928966 | *Marmota himalayana* | CHN | 2013 |
| KY928967 | *Marmota himalayana* | CHN | 2013 |
| KY928968 | *Marmota himalayana* | CHN | 2013 |
| KY928969 | *Marmota himalayana* | CHN | 2013 |
| KY928970 | *Marmota himalayana* | CHN | 2013 |
| KY928971 | *Marmota himalayana* | CHN | 2013 |
| KY928972 | *Marmota himalayana* | CHN | 2013 |
| KY928973 | *Marmota himalayana* | CHN | 2013 |
| KY928974 | *Marmota himalayana* | CHN | 2013 |
| KY928975 | *Marmota himalayana* | CHN | 2013 |
| KY928976 | *Marmota himalayana* | CHN | 2013 |
| KY928977 | *Marmota himalayana* | CHN | 2013 |
| KY928978 | *Marmota himalayana* | CHN | 2013 |
| KY928979 | *Marmota himalayana* | CHN | 2013 |
| KY928980 | *Marmota himalayana* | CHN | 2013 |
| KY928981 | *Marmota himalayana* | CHN | 2013 |
| KY928982 | *Marmota himalayana* | CHN | 2013 |
| KY928983 | *Marmota himalayana* | CHN | 2013 |
| KY928984 | *Marmota himalayana* | CHN | 2013 |
| KY928985 | *Marmota himalayana* | CHN | 2013 |
| KY928986 | *Marmota himalayana* | CHN | 2013 |
| KY928987 | *Marmota himalayana* | CHN | 2013 |
| KY928988 | *Marmota himalayana* | CHN | 2013 |
| KY928989 | *Marmota himalayana* | CHN | 2013 |
| KY928991 | *Marmota himalayana* | CHN | 2013 |
| KY928992 | *Marmota himalayana* | CHN | 2013 |
| KY928993 | *Marmota himalayana* | CHN | 2013 |
| KY928994 | *Marmota himalayana* | CHN | 2013 |
| KY928995 | *Marmota himalayana* | CHN | 2013 |
| KY928996 | *Marmota himalayana* | CHN | 2013 |
| KY928997 | *Marmota himalayana* | CHN | 2013 |
| KY928998 | *Marmota himalayana* | CHN | 2013 |
| KY928999 | *Marmota himalayana* | CHN | 2013 |
| KY929000 | *Marmota himalayana* | CHN | 2013 |
| KY929001 | *Marmota himalayana* | CHN | 2013 |

| | | | |
|---|---|---|---|
| KY929002 | *Marmota himalayana* | CHN | 2013 |
| KY929003 | *Marmota himalayana* | CHN | 2013 |
| KY929005 | *Marmota himalayana* | CHN | 2013 |
| KY929006 | *Marmota himalayana* | CHN | 2013 |
| KY929007 | *Marmota himalayana* | CHN | 2013 |
| KY929008 | *Marmota himalayana* | CHN | 2013 |
| KY929009 | *Marmota himalayana* | CHN | 2013 |
| KY929010 | *Marmota himalayana* | CHN | 2013 |
| KY929011 | *Marmota himalayana* | CHN | 2013 |
| KY929012 | *Marmota himalayana* | CHN | 2013 |
| LC110352 | *Mus musculus* | JPN | 2015 |
| LC337994 | *Camelus dromedaries* | ARE | 2013 |
| LC337995 | *Camelus dromedaries* | ARE | 2013 |
| LC337996 | *Camelus dromedaries* | ARE | 2013 |
| LC337997 | *Camelus dromedaries* | ARE | 2013 |
| LC337998 | *Camelus dromedaries* | ARE | 2013 |
| LC338000 | *Camelus dromedaries* | ARE | 2013 |
| MG010885 | *Macaca mulatta* | TCD | 2011 |
| MG010886 | *Macaca mulatta* | TCD | 2011 |
| MG010887 | *Macaca mulatta* | TCD | 2011 |
| MG010888 | *Macaca mulatta* | TCD | 2011 |
| MG010889 | *Macaca mulatta* | TCD | 2011 |
| MG010890 | *Macaca mulatta* | TCD | 2011 |
| MG010891 | *Macaca mulatta* | TCD | 2011 |
| MG010892 | *Macaca mulatta* | TCD | 2011 |
| MG010893 | *Macaca mulatta* | TCD | 2011 |
| MG010894 | *Macaca mulatta* | TCD | 2011 |
| MG010895 | *Macaca mulatta* | TCD | 2011 |
| MG010896 | *Macaca mulatta* | TCD | 2011 |
| MG010898 | *Macaca mulatta* | TCD | 2011 |
| MG190028 | *Roe deer* | SVN | 2014 |
| MG600063 | *Parupeneus cyclosto-mus* | CHN | NA |
| MG846392 | *Gallus gallus* | BRA | 2015 |
| MG846393 | *Gallus gallus* | BRA | 2015 |
| MG846395 | *Gallus gallus* | BRA | 2015 |
| MG846396 | *Gallus gallus* | BRA | 2015 |
| MG846397 | *Gallus gallus* | BRA | 2015 |
| MG846398 | *Gallus gallus* | BRA | 2015 |
| MG846399 | *Gallus gallus* | BRA | 2015 |
| MG846400 | *Gallus gallus* | BRA | 2015 |
| MH327933 | *Gallus gallus* | HUN | 2011 |
| MH425579 | *Gallus gallus* | HUN | 2013 |
| MH425580 | *Gallus gallus* | HUN | 2013 |
| MH425581 | *Gallus gallus* | HUN | 2013 |
| MH425582 | *Gallus gallus* | HUN | 2013 |
| MH425583 | *Gallus gallus* | HUN | 2013 |
| MH453876 | *Australian Shelduck* | AUS | 2012 |
| MH453877 | *Australian Shelduck* | AUS | 2012 |
| MK204399 | *Anas gracilis* | AUS | 2017 |
| MK204419 | *Malacorhynchus mem-branaceus* | AUS | 2017 |
| MK305309 | *Homo sapiens* | AGO | 2018 |
| MK378829 | *Sus scrofa* | CHN | 2004 |
| MK378842 | *Sus scrofa* | CHN | 2004 |
| MK378864 | *Sus scrofa* | CHN | 2004 |
| MN692670 | *Macaca fascicularis* | THA | 2017 |
| MT846991 | *Pantholops hodgsonii* | CHN | 2014 |
| MT846992 | *Pantholops hodgsonii* | CHN | 2014 |

| | | | |
|---|---|---|---|
| MT846993 | *Pantholops hodgsonii* | CHN | 2014 |
| MT846994 | *Pantholops hodgsonii* | CHN | 2014 |
| MT846995 | *Pantholops hodgsonii* | CHN | 2014 |
| MT846996 | *Pantholops hodgsonii* | CHN | 2014 |
| MT846997 | *Pantholops hodgsonii* | CHN | 2014 |
| MT846998 | *Pantholops hodgsonii* | CHN | 2014 |
| MT846999 | *Pantholops hodgsonii* | CHN | 2014 |
| NC-029801 | *Sus scrofa* | ITA | 2004 |
| NC-034160 | *Zalophus californianus* | AGO | 2008 |
| NC-040438 | *Gallus gallus* | HUN | 2011 |
| NC-040752 | *Roe deer* | SVN | 2014 |
| KC692367 | *Vulpes vulpes* | NLD | 2012 |
| KY502844 | *Gorilla gorilla* | COD | 2015 |
| AB186897 | *NA* | THA | NA |

**Table S3.** Genetic distances based on complete RDRP coding sequences of all the lineages assessed within the PBV species 1**.** Values above the diagonal represent the standard error, values below the diagonal represent the p-distance values obtained using MEGAX and 1000 bootstrap replicates. Values shadowed in gray are below the cut-off determined to establish different genotypes (see Figure 5A), thus five genotypes for PBV1 are defined after regrouping and recalculating the distances (see Figure 5A).

| **Gen** | **1.1** | **1.2** | **1.3** | **1.4** | **1.5** | **1.6** | **1.7** | **1.8** | **1.9** | **1.10** | **1.11** | **1.12** |
|---|---|---|---|---|---|---|---|---|---|---|---|---|
| **1.1** | | *0.0169* | *0.0169* | *0.0180* | *0.0168* | *0.0170* | *0.0166* | *0.0178* | *0.0228* | *0.0197* | *0.0169* | *0.0166* |
| **1.2** | 0.3265 | | *0.0158* | *0.0177* | *0.0162* | *0.0159* | *0.0147* | *0.0188* | *0.0217* | *0.0183* | *0.0159* | *0.0169* |
| **1.3** | 0.3139 | 0.3110 | | *0.0161* | *0.0166* | *0.0162* | *0.0169* | *0.0197* | *0.0239* | *0.0188* | *0.0174* | *0.0164* |
| **1.4** | 0.3624 | 0.3678 | 0.3490 | | *0.0166* | *0.0171* | *0.0169* | *0.0186* | *0.0213* | *0.0190* | *0.0162* | *0.0162* |
| **1.5** | 0.3713 | 0.3744 | 0.3649 | 0.3896 | | *0.0155* | *0.0157* | *0.0166* | *0.0208* | *0.0176* | *0.0158* | *0.0162* |
| **1.6** | 0.3558 | 0.3560 | 0.3545 | 0.3833 | 0.3772 | | *0.0155* | *0.0190* | *0.0208* | *0.0175* | *0.0150* | *0.0160* |
| **1.7** | 0.3468 | 0.3485 | 0.3515 | 0.3775 | 0.3729 | 0.3507 | | *0.0187* | *0.0224* | *0.0176* | *0.0161* | *0.0161* |
| **1.8** | 0.3505 | 0.3406 | 0.3344 | 0.3714 | 0.3816 | 0.3628 | 0.3627 | | *0.0249* | *0.0202* | *0.0154* | *0.0158* |
| **1.9** | 0.4327 | 0.4107 | 0.4290 | 0.4489 | 0.4541 | 0.4262 | 0.4290 | 0.4154 | | *0.0211* | *0.0190* | *0.0172* |
| **1.10** | 0.4180 | 0.4145 | 0.4150 | 0.4287 | 0.4128 | 0.4140 | 0.4155 | 0.4086 | 0.4475 | | *0.0175* | *0.0162* |
| **1.11** | 0.4976 | 0.4973 | 0.5029 | 0.5040 | 0.5004 | 0.4954 | 0.5046 | 0.5098 | 0.5113 | 0.5139 | | *0.0152* |
| **1.12** | 0.4824 | 0.4895 | 0.4897 | 0.4860 | 0.4892 | 0.4881 | 0.4930 | 0.4959 | 0.5119 | 0.5030 | 0.5192 | |

**Table S4.** Genetic distances based on complete RDRP coding sequences of all the lineages assessed within the PBV species 2**.** Values above the diagonal represent the standard error, values below the diagonal represent the p-distance values obtained using MEGAX and 1000 bootstrap replicates. Values shadowed in gray are below the cut-off determined to establish different genotypes (see Figure 5B), thus eight genotypes for PBV2 are defined after regrouping and recalculating the distances (see Figure 5B).

| **Gen** | **2.1** | **2.2** | **2.3** | **2.4** | **2.5** | **2.6** | **2.7** | **2.8** | **2.9** | **2.10** | **2.11** |
|---|---|---|---|---|---|---|---|---|---|---|---|
| **2.1** | | *0.0166* | *0.0182* | *0.0193* | *0.0199* | *0.0205* | *0.0175* | *0.0219* | *0.0219* | *0.0224* | *0.0230* |
| **2.2** | 0.3799 | | *0.0197* | *0.0199* | *0.0199* | *0.0206* | *0.0175* | *0.0220* | *0.0233* | *0.0232* | *0.0231* |
| **2.3** | 0.4070 | 0.4496 | | *0.0195* | *0.0204* | *0.0213* | *0.0187* | *0.0235* | *0.0250* | *0.0242* | *0.0249* |
| **2.4** | 0.4336 | 0.4353 | 0.3728 | | *0.0209* | *0.0226* | *0.0190* | *0.0241* | *0.0251* | *0.0244* | *0.0250* |
| **2.5** | 0.5195 | 0.4958 | 0.5066 | 0.4623 | | *0.0196* | *0.0177* | *0.0227* | *0.0235* | *0.0235* | *0.0249* |
| **2.6** | 0.4574 | 0.4512 | 0.4485 | 0.4716 | 0.3590 | | *0.0192* | *0.0254* | *0.0246* | *0.0247* | *0.0264* |
| **2.7** | 0.4827 | 0.4967 | 0.4776 | 0.4960 | 0.5113 | 0.4745 | | *0.0098* | *0.0208* | *0.0223* | *0.0212* |
| **2.8** | 0.4765 | 0.4945 | 0.4775 | 0.5049 | 0.5132 | 0.4667 | 0.4316 | | *0.0274* | *0.0281* | *0.0270* |
| **2.9** | 0.4413 | 0.4719 | 0.4228 | 0.4235 | 0.4486 | 0.4207 | 0.5060 | 0.4930 | | *0.0296* | *0.0278* |
| **2.10** | 0.4126 | 0.5092 | 0.4892 | 0.5018 | 0.5292 | 0.5007 | 0.5110 | 0.5211 | 0.4632 | | *0.0284* |
| **2.11** | 0.4130 | 0.4357 | 0.5092 | 0.4835 | 0.4180 | 0.5060 | 0.5130 | 0.5246 | 0.4140 | 0.4000 | |

**Table S5.** Genetic distances based on complete RDRP coding sequences of all the lineages assessed within the PBV species 3**.** Values above the diagonal represent the standard error, values below the diagonal represent the p-distance values obtained using MEGAX and 1000 bootstrap replicates. Values shadowed in gray are below the cut-off determined to establish different genotypes (see Figure 5C), thus three genotypes for PBV3 are defined after regrouping and recalculating the distances.

| **Gen** | **3.1** | **3.2** | **3.3** | **3.4** | **3.5** | **3.6** |
|---|---|---|---|---|---|---|
| **3.1** | | *0.0215* | *0.0253* | *0.0258* | *0.0258* | *0.0240* |
| **3.2** | 0.3103 | | *0.0248* | *0.0249* | *0.0245* | *0.0232* |
| **3.3** | 0.4216 | 0.4290 | | *0.0231* | *0.0253* | *0.0257* |
| **3.4** | 0.3981 | 0.4027 | 0.2557 | | *0.0242* | *0.0261* |
| **3.5** | 0.4246 | 0.4327 | 0.3063 | 0.2500 | | *0.0262* |
| **3.6** | 0.4249 | 0.4133 | 0.4537 | 0.4260 | 0.4569 | |
